# Genome-wide association study of brain biochemical phenotypes reveals distinct genetic architecture of Alzheimer’s disease related proteins

**DOI:** 10.1186/s13024-022-00592-2

**Published:** 2023-01-07

**Authors:** Stephanie R. Oatman, Joseph S. Reddy, Zachary Quicksall, Minerva M. Carrasquillo, Xue Wang, Chia-Chen Liu, Yu Yamazaki, Thuy T. Nguyen, Kimberly Malphrus, Michael Heckman, Kristi Biswas, Kwangsik Nho, Matthew Baker, Yuka A. Martens, Na Zhao, Jun Pyo Kim, Shannon L. Risacher, Rosa Rademakers, Andrew J. Saykin, Michael DeTure, Melissa E. Murray, Takahisa Kanekiyo, for the Alzheimer’s Disease Neuroimaging Initiative, Dennis W. Dickson, Guojun Bu, Mariet Allen, Nilüfer Ertekin-Taner

**Affiliations:** 1 Department of Neuroscience, Mayo Clinic, 4500 San Pablo Road, Jacksonville, FL 32224 USA; 2 Department of Quantitative Health Sciences, Mayo Clinic, Jacksonville, FL USA; 3 Indiana Alzheimer Disease Center, Indiana University School of Medicine, Indianapolis, IN USA; 4 Department of Radiology and Imaging Sciences, Indiana University School of Medicine, Indianapolis, IN USA; 5 School of Informatics and Computing, Indiana University School of Medicine, Indianapolis, IN USA; 6 VIB-UA Center for Molecular Neurology, VIB, University of Antwerp, Antwerp, Belgium; 7 Department of Medical and Molecular Genetics, Indiana University School of Medicine, Indianapolis, IN USA; 8 Department of Neurology, Mayo Clinic, 4500 San Pablo Road, Birdsall 3, Jacksonville, FL 32224 USA

**Keywords:** Alzheimer’s, Genetics, APOE, Amyloid, Tau, Association, Brain, GWAS, Neuroscience, Diseases, Biochemistry

## Abstract

**Background:**

Alzheimer’s disease (AD) is neuropathologically characterized by amyloid-beta (Aβ) plaques and neurofibrillary tangles. The main protein components of these hallmarks include Aβ40, Aβ42, tau, phosphor-tau, and APOE. We hypothesize that genetic variants influence the levels and solubility of these AD-related proteins in the brain; identifying these may provide key insights into disease pathogenesis.

**Methods:**

Genome-wide genotypes were collected from 441 AD cases, imputed to the haplotype reference consortium (HRC) panel, and filtered for quality and frequency. Temporal cortex levels of five AD-related proteins from three fractions, buffer-soluble (TBS), detergent-soluble (Triton-X = TX), and insoluble (Formic acid = FA), were available for these same individuals. Variants were tested for association with each quantitative biochemical measure using linear regression, and GSA-SNP2 was used to identify enriched Gene Ontology (GO) terms. Implicated variants and genes were further assessed for association with other relevant variables.

**Results:**

We identified genome-wide significant associations at seven novel loci and the *APOE* locus. Genes and variants at these loci also associate with multiple AD-related measures, regulate gene expression, have cell-type specific enrichment, and roles in brain health and other neuropsychiatric diseases. Pathway analysis identified significant enrichment of shared and distinct biological pathways.

**Conclusions:**

Although all biochemical measures tested reflect proteins core to AD pathology, our results strongly suggest that each have unique genetic architecture and biological pathways that influence their specific biochemical states in the brain. Our novel approach of deep brain biochemical endophenotype GWAS has implications for pathophysiology of proteostasis in AD that can guide therapeutic discovery efforts focused on these proteins.

**Supplementary Information:**

The online version contains supplementary material available at 10.1186/s13024-022-00592-2.

## Background

Alzheimer’s disease (AD) is a progressive neurodegenerative disorder, neuropathologically characterized by the accumulation of amyloid beta (Aβ) plaques and neurofibrillary tangles (NFT) in the brain [1, 2]. While AD neuropathology broadly follows characteristic patterns, heterogeneity in the composition, location and burden of the two primary lesions has been reported across post-mortem datasets [3–8]. The main component of insoluble amyloid plaques is Aβ42, while Aβ40 is often found deposited in the brain cerebrovasculature called cerebral amyloid angiopathy (CAA) [8]. Aβ is generated by the normal cleavage of the amyloid-beta precursor protein (APP), which then can oligomerize and form extracellular deposits [9, 10]. Some mutations in the *APP* gene cause a familial early-onset form of AD through modification of APP cleavage resulting in an increase in Aβ42 production [11]. Increased tau levels are also observed in AD, along with abnormal hyperphosphorylation leading to aggregation into insoluble NFT within the cell body [2, 12]. Under normal conditions in the brain the soluble tau protein is found relatively un-phosphorylated and bound to microtubules for stabilization [13–15]. Previous genetic studies of late-onset AD (LOAD) have found variants associated with the risk of developing AD; the most significant of which is the well-established *APOE-*ε4 allele [16–18]. *APOE* encodes apolipoprotein E (APOE) which functions mainly in lipid transport, but is also known to play a role in Aβ metabolism and its insoluble forms are often found co-deposited with Aβ plaques [19]. Beyond insoluble deposits of amyloid and tau species, soluble and membrane-associated biochemical states of these proteins have also been associated with AD-related phenotypes. In the temporal cortex, soluble levels of Aβ40 and Aβ42 are significantly elevated in AD compared to controls and Aβ40 levels positively correlate with disease duration [20, 21]. Membrane-associated forms of Aβ show a significant positive correlation with Aβ positron emission tomography (PET) imaging in AD, while cortical Aβ42 levels have been reported to correlate with worse clinical severity and increased rate of cognitive decline [20, 21]. Moreover, it has been shown that when tau interacts with the plasma membrane, the propensity for fibrillization increases, and within the context of AD, variability in soluble tau has been shown to occur in the presence of Aβ pathology but before significant NFT pathology [22–31]. Apart from *APOE-*ε4, genetic risk factors associated with different brain biochemical states of distinct proteins core to AD pathology have yet to be identified and characterized [32].

**Fig. 1:**
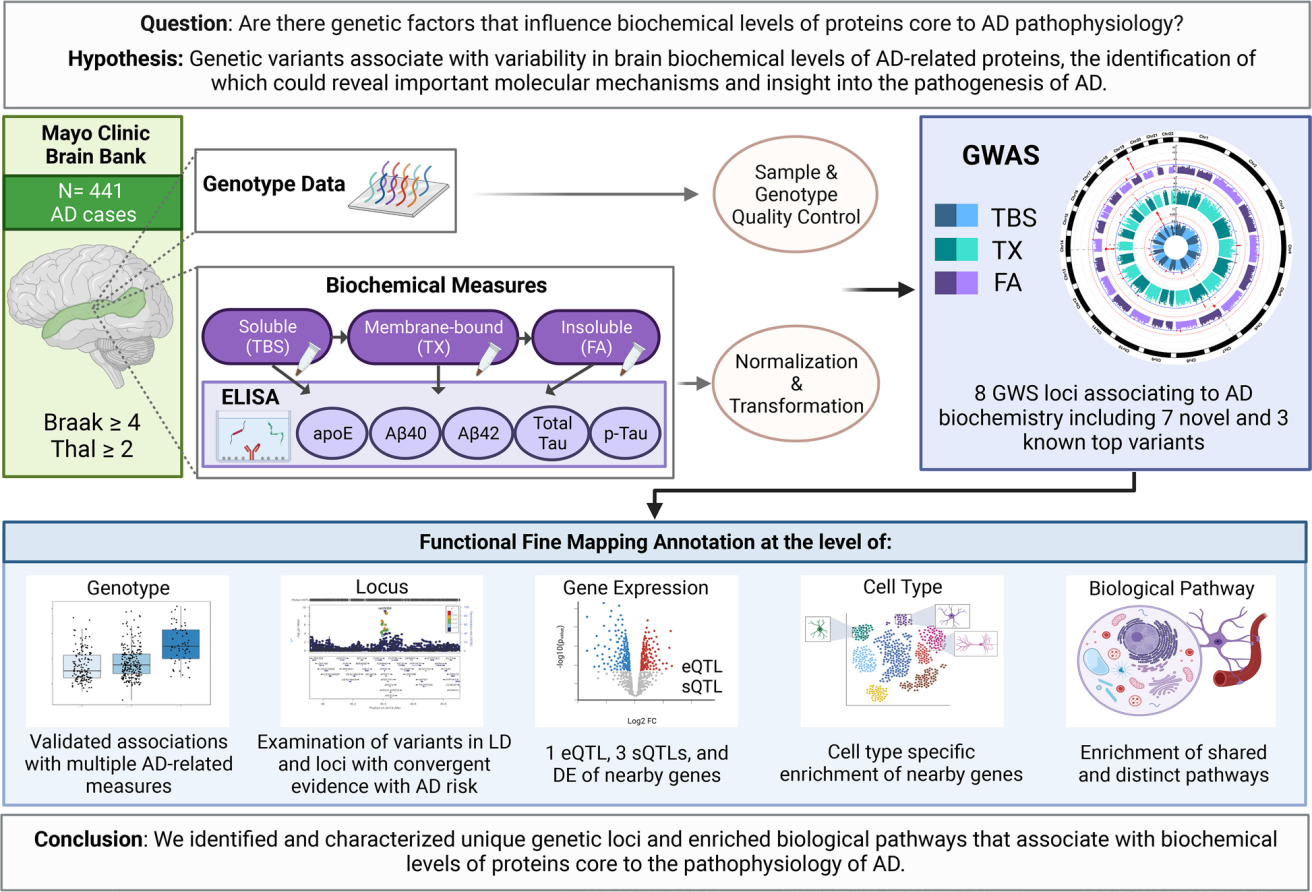
Graphical Abstract. Graphical depiction of this study: AD = Alzheimer’s Disease, N = Number, Aβ = amyloid beta, p-Tau = phosphorylated tau, TBS = Tris Buffered Saline, TX = Triton-X, FA = Formic Acid, GWS = Genome-wide significant, LD = Linkage Disequilibrium, eQTL = expression quantitative trait locus, sQTL = splicing quantitative trait locus, DE = Differential expression. Created with BioRender.com

We hypothesize that important insights into the pathogenesis of AD may be gained by identifying genetic variants associated with variability in brain levels of AD-related protein endophenotypes including Aβ40, Aβ42, tau, phosphorylated tau (p-Tau) and APOE. Furthermore, different biochemical states (soluble, membrane, and insoluble) of AD-related proteins may have distinct genetic variants that influence their levels within the brain. Such findings may provide key insights into production or clearance pathways for these disease-associated proteins, leading to novel therapeutic targets or biomarkers. To investigate this, we utilized genetic and biochemical measures collected from the temporal cortex of 441 post-mortem AD cases. We performed a genome-wide association study (GWAS) for levels of all five proteins, collected from three biochemical states in the brain (Fig. 1). Our findings reveal novel genetic loci and highlight the unique genetic architecture for specific biochemical states of AD-related protein endophenotypes. This study establishes deep brain biochemical endophenotype GWAS as a novel approach to dissect the biochemical heterogeneity of AD proteins which is essential to fine-tune therapeutic efforts targeting these proteins.

## Methods

### Brain samples

Post-mortem temporal cortex samples included in this study were a part of the Mayo Clinic AD-CAA (MC-CAA) study on the AD-knowledge portal (https://adknowledgeportal.synapse.org, see data sharing). All samples had a confirmed AD neuropathological diagnosis, a Braak stage ≥ four, Thal phase ≥ three, and were from non-Hispanic White decedents of Northern European descent. In total, 441 samples had both genome-wide genotyping data and biochemical measures available for analyses. This study was approved by the appropriate Mayo Clinic Institutional Review Board.

### Neuropathology

Braak stage, Thal phase, and CAA scores were measured by the Mayo Clinic Brain Bank using previously established protocols [32–36]. Intermediate Braak stages were grouped with the next lowest stage as follows, stage 3.5 is 3, 4.5 is 4, and 5.5 is 5 as detailed previously [32, 37].

### Biochemical measures

Biochemical measures from 441 of the 469 superior temporal cortex brain samples previously described [32] were utilized for this study based on the availability of genome-wide genotypes. Biochemical measures include five AD-related proteins (APOE, Aβ40, Aβ42, tau, and phospho-tau (Thr231)) from three tissue fractions. Briefly, supernatant fractions were collected after three sequential buffer treatments of tissue homogenate and resulting pellets: first with tris-buffered saline buffer (TBS), second with detergent (TBS/1% Triton X) buffer (TX), and finally with formic acid buffer (FA), representing soluble, lipid-membrane and insoluble biochemical fractions. AD-related biochemical measures were quantified in each fraction via ELISA and normalized against total protein quantities. All biochemical measures were transformed by either the natural log or square root to achieve an approximately normal distribution including the Aβ40/42 ratio (Fig. S1). In the subset of 441 samples analyzed, we evaluated the association of the AD-related proteins within and among these tissue fractions through pairwise correlation and found similar results as reported previously [32] (Data not shown).

### Genotyping

DNA was isolated from brain tissue using the AutoGen245T instrument according to manufacturer’s protocols, incubated with two μl (4 mg.ml) RNAseA solution (Qiagen, Germany) and stored at -80 °C until use. Genome-wide genotypes from 477 samples were previously collected [37] using the Infinium Omni2.5 Exome8 v1.3 genotyping array and results were exported using the Illumina GenomeStudio software v1.9.4. Data was formatted into PLINK (v1.9) files [38, 39] (lgen, fam, and map) and quality control of the samples and genotypes was performed, as described in detail elsewhere [37]. Four hundred sixty samples passed quality control (QC), of which 441 also had biochemical measures [32]. Variants passing quality control (*N* = 1,383,987) were imputed to the haplotype reference consortium (HRC) panel [40, 41] and those with an imputation quality *R*^2^ ≥ 0.7 and MAF ≥ 2% were kept, yielding a total of 6,726,078 variants for analysis. Genotype dosages were converted to hard calls when needed with uncertainty > 0.1 set to missing. Minor allele frequencies and Hardy-Weinberg *p*-values were calculated for all reported variants using dosages in PLINK [38, 39].

Genotypes for key variants, or their proxies (*r*^2^ = 1, D′ = 1 in 1000 Genomes EUR), were validated by Taqman genotyping or Sanger sequencing following the manufacturer’s protocols. These assays were also used to collect genotypes from an additional 1564 Mayo Clinic Brain Bank (MCBB) samples with available DNA to enable assessment with AD-related neuropathology measures of Braak stage, Thal phase, neuropathological diagnosis of AD, and age at death (combined *N* = 2005, Table S7). These combined 2005 AD samples are collectively referred to as the Mayo Clinic Brain Bank Expansion Cohort and are non-overlapping with the AMP-AD Mayo Clinic cohort described below. Taqman genotyping assays were performed using 10 ng of dried-down DNA and the QuantStudio 7 Flex system (Thermo Fisher Scientific, USA) for 7 variants (Table S14). Genotypes for *APOE*-rs429358 were previously collected using Taqman assays and queried from a database. One variant, *STRN4*-rs34805055, failed genotyping assay design and had no viable proxies, so Sanger sequencing was performed to validate all minor allele carrier samples. Sequencing was done on the ABI 3730 Genetic Analyzer instrument (Thermo Fisher Scientific, USA) following PCR amplification with the following primer pair: forward (5′- GGAAAGCAGCTCTGATAC) and reverse (5′- CGCATTCTGAGTCTCTG) (Integrated DNA Technologies, USA).

### AMP-AD datasets

The Mayo RNAseq study [42], The Mount Sinai Brain Bank (MSBB) study [43] and The Religious Orders Study and Memory and Aging Project (ROSMAP) Study [44], were obtained from the AD-knowledge portal (https://adknowledgeportal.synapse.org). Available brain tissue RNAseq data, whole-genome genotypes, and neuropathological variables collected from these three studies were downloaded and used for fine-mapping of GWS loci and association analyses. The inclusion of these large, well-characterized, and harmonized datasets from the AMP-AD consortia with complementary multi-omics datasets allow us to investigate and characterize GWS variants and loci across multiple regions of the brain for associations with AD-related phenotypes and brain gene expression levels in these cohorts. The RNA-seq data consists of seven datasets, two from Mayo Clinic (TCX and CER), four from MSBB (BM10, BM22, BM36, and BM44), and one from ROS-MAP (DLPFC) and previously underwent consensus reprocessing (AMP-AD, RNAseq Harmonization Study) [45]. Additional QC and diagnosis harmonization of these datasets based on neuropathological measures retrieved from individual metadata files are described in detail elsewhere [37].

In all cohorts, diagnosis was determined primarily by neuropathology made by experienced neuropathologists. The following criteria were used for diagnoses: AMP-AD Mayo dataset AD patients had a Braak stage ≥4 while nonADs had a Braak stage ≤3. AMP-AD MSBB dataset AD patients had a Braak stage ≥4 and CERAD score ≥ 2 while nonADs had Braak stage ≤3 and CERAD score ≤ 1. AMP-AD ROS-MAP dataset AD patients had a Braak stage ≥4 and CERAD score ≤ 2 while nonADs had Braak stage ≤3 and CERAD score ≥ 3. Of note, MSBB and ROS-MAP used different CERAD definitions. In ROS-MAP, CERAD score (1-4) was based on semiquantitative estimates of neuritic plaque density in one or more neocortical regions following recommendations by the Consortium to Establish a Registry for Alzheimer’s Disease (CERAD) protocol [46]. In MSBB, a CERAD 1 = Normal, 2 = Definite AD, 3 = probable AD, and 4 = possible AD. In ROSMAP, 1 = Definite AD, 2 = probable AD, 3 = Possible AD, and 4 = No AD.

Whole genome sequencing (WGS) data from each AMP-AD cohort was processed separately using an automated pipeline at the New York Genome Center. 150 bp paired-end reads were aligned to GRCh37 human reference genome using Burrows-Wheeler Aligner [47] (BWA-MEM v0.7.08). After marking duplicates with Picard tools [48] (v1.83) and local read alignment around indels, base quality score recalibration (BQSR) was performed using Genome Analysis Toolkit [49] (GATK v 3.4.0). Variant calling and joint genotyping were performed using GATK’s HaplotypeCaller (GATK v3.4.0) and GenotypeGVCFs (GATK v.3.5), respectively, to generate a multisample VCF file for each dataset. Variant quality was assessed using GATK’s variant quality score recalibration (VQSR) tool. After obtaining multi-sample VQSR-ed VCFs for each individual study from the AD knowledge portal (see data sharing), genotypes were imported into PLINK [38, 39] (v1.9) for additional sample and variant QC using an in-house next-generation sequencing QC pipeline. Bi-allelic autosomal variants that pass VQSR FILTER, having a genotyping rate > =98% and a minor allele frequency > = 2%, and a Bonferroni adjusted HWE *p*-value in controls > 0.05 were retained for downstream analysis. Variants within high variability regions of the genome that can lead to spurious associations were excluded. Samples with a call rate > =98%, sex concordant with clinical information as evaluated using the inbreeding coefficient of the X-chromosome (males > = 0.7, females<=0.3) and a heterozygosity estimate within 3 standard deviations (SD) of mean were retained. Relatedness among samples within each cohort was evaluated using KING [50] robust and only one sample from each pair or family of samples related to the third degree (kinship estimate > = 0.0442) was retained. Population substructure was evaluated using Eigenstrat [51, 52] and outliers beyond 6 SD of the top 10 principal components were removed over five iterations while refitting PCs after each iteration. After performing sample and variant QC within each cohort, data from all three datasets was merged and relatedness and population substructure were re-evaluated to exclude related samples and population outliers across all three datasets. In summary, unrelated samples of relatively homogeneous non-Hispanic White ancestry that met the aforementioned sample and variant QC metrics were retained for downstream analyses.

### Alzheimer’s Disease Neuroimaging Initiative (ADNI) dataset

The ADNI was launched in 2003 as a public-private partnership, led by Principal Investigator Michael W. Weiner, MD [53]. The primary goal of ADNI has been to test whether serial magnetic resonance imaging (MRI), positron emission tomography (PET), other biological markers, and clinical and neuropsychological assessment can be combined to measure the progression of mild cognitive impairment (MCI) and early Alzheimer’s disease [54]. Inclusion and exclusion criteria, clinical and neuroimaging protocols, and other information about ADNI can be found at www.adni-info.org. Data used in the preparation of this article were obtained from the Alzheimer’s Disease Neuroimaging Initiative (ADNI) database (adni.loni.usc.edu). Demographic information, apolipoprotein E (APOE) and genome, Pre-processed [^18^F] Florbetapir PET scans, plasma total Aβ42, and plasma tau phosphorylated at the threonine 181 (p-tau) data, and clinical information are available and were downloaded from the ADNI data repository (www.loni.usc.edu/ADNI/). [^18^F] Florbetapir PET scans were intensity-normalized using a whole cerebellum reference region to create SUVR images [55]. CSF biomarkers (Amyloid-β 1-42 peptide (Aβ1-42), total tau (t-tau), and p-tau) were generated by the validated and highly automated Roche Elecsys® electrochemiluminescence immunoassays and the same reagent lot for each of these three biomarkers [56]. The ADNI participants were genotyped using several Illumina genotyping platforms. After quality control procedures for participants and SNPs, un-genotyped SNPs for non-Hispanic participants of European ancestry were imputed separately in each platform using Markov Chain Haplotyping with the Haplotype Reference Consortium data as a reference panel [57].

### Statistical analysis

Power calculations were performed in R (v4.0.2) with the genpwr package. For a sample size of 441, and an alpha = 5E-08, we have 80% power to detect effect sizes of 0.42 and 0.96 when the minor allele frequency (MAF) is 0.5 and 0.05, respectively. Principal component analysis with automatic outlier exclusion was performed using Eigenstrat [51, 52], no population outliers were identified. PLINK was utilized to perform PCA without outlier exclusion to examine samples in this cohort relative to 1000G superpopulations. PCA plots were generated using the ploty_ly() package in R (v3.6) (Fig. S5).

PLINK (v2.00a2LM) was used to perform genome-wide association tests for variant dosage associations with each biochemical measure adjusting for age, sex and the first three population principal components (PCs). When specified, the *APOE-*ε2 and -ε4 alleles as well as *APOE* diplotypes 23, 24, 34, and 44 (33 was set as the reference) were included as covariates. There were no *APOE* 22 samples. Genomic inflation values (λ) were calculated in R (v3.6.2) for each biochemical measure with and without adjustment of *APOE-*ε2 and -ε4 alleles (Fig. S6); there was no evidence for genomic inflation (0.97 < λ < 1.02). QQ plots were generated in R (v3.6) with the ggplot package (Fig. S6). A genome-wide significance (GWS) threshold was calculated based on the number of independent variants in our dataset which also accounts for the inclusion of low frequency variants [58]. Quality control of the imputed variants to the HRC reference panel yielded a total of 6,726,078 variants. To calculate the number of independent variants, we used the ‘--indep-pairwise’ flag in PLINK with the window size set to 50 kb, step size set to 5, and *R*^2^ threshold set to 0.8. After this pruning, we had 1,679,420 independent variants which we used to calculate our study GWS threshold of 2.98 × 10^-8^ (0.05/1,679,420). To determine if GWS associations were independent from the effect of the *APOE-ε*4 allele, conditional analyses were run in PLINK 2.0 implementing the '--condition' command in a linear regression model conditioning on the *APOE-*ε4 tagging variant rs429358 and adjusting for age, sex, and PCs1-3. LD analysis was performed using PLINK (± 1 Mb, D′ ≥ 0.8 and *r*^2^ ≥ 0.2). The estimated proportion of biochemical measure variance explained by the GWS index SNPs was based on the *R*^2^ calculated through linear regression models regressing appropriate index SNPs on each biochemical measure.

Variants were tested for association with AD neuropathology and other related measures in the AMP-AD and expanded MCBB datasets using multi-variable regression analysis. Braak stage and Thal phase were assessed with ordinal regression in R (v4.0.2), diagnosis with logistic regression in PLINK, and age at death with linear regression in PLINK (v2.00a2LM). Samples with age at death greater than 90 years in the MCBB were redacted to 90 to parallel protocols of the AMP-AD datasets. All models included sex as a covariate, and age at death, *APOE-ε*2, and *APOE-ε*4 when appropriate or specified. Meta- analysis was performed in R (v4.0.2) with the *meta* function for both fixed and random effects models.

The AMP-AD datasets were used to assess of each locus with brain gene expression. Differential expression analysis between diagnosis (AD case or control) and normalized gene expression levels was performed using linear regression implemented in R (v3.5.2) adjusting for age at death, sex, RNA integrity number (RIN), and sequencing batch. eQTL analysis was performed by testing variant association with CQN gene expression levels in a linear mixed model using the *lme4* package in R (v3.5.2) adjusting for diagnosis, sex, age at death, RIN, tissue source, and the first three PCs, with the flow cell added as a random effects variable.

Variants were investigated in the ADNI dataset by performing a linear regression association analysis of variants with AD endophenotypes using additive genetic models adjusted for age and sex with or without *APOE*ɛ4 carrier status inclusion as an additional covariate.

Colocalization analyses for GWS loci with evidence of a significant QTL was performed. For loci with eQTL evidence from AMP-AD datasets, we used the *coloc*() R package [59, 60] with summary GWAS and QTL statistics as inputs +/− 500 Kb from the index variant and linkage disequilibrium estimates from the 1000 Genomes EUR dataset. Single causal variant colocalization was investigated with the *coloc.abf* function while multiple causal variants were investigated with the *runsusie* and *susie.coloc* functions [61] in *coloc* which uses the Sum of Single Effects (SuSiE) regression framework [60]. The suggested decision rule of hypothesis 4 (H4) having a posterior probability (PP) value > 0.9 was used to indicate colocalization. For loci with QTL evidence from GTEx, the ezQTL webserver tool made available through the NIH Division of Cancer Epidemiology and Genetics at the National Cancer Institute (analysistools.cancer.gov/ezqtl/#/home) [62] was utilized to investigate single causal variant colocalization with HyPrColoc [63] and multiple causal variant colocalization with eCAVIAR [64]. GWAS summary statistics were lifted from hg19 to hg38 using the UCSC chain file (hg19ToHg38, downloaded from genome.ucsc.edu/ on 11/04/2022) and the R package *rtracklayer::liftOver*(). GTEx v8 summary data and 1000 Genomes EUR LD data were precompiled by ezQTL.

### Pathway analysis

Gene set enrichment analysis was performed for each GWAS result with GSA-SNP2 software [65] against the MSigDb c5.all.v5.2 database [66, 67]. Options selected include European race, GRCh37(hg19) padding build, and pathway size window of 10-200. GSA-SNP2 results were matched with Gene Ontology (GO) [68, 69] term IDs using an in-house script. Significant GO terms and *p*-values were input into REViGO [70] to summarize significantly enriched pathways. REViGO settings were as follows: medium (0.7) allowed similarity, *Homo sapiens* (Gene Ontology Jan 2017) database, and SimRel semantic similarity measure. Summary bar charts were created in R (v3.6.2) with ggplot by taking reduced pathway groups from the REViGO outputs and the most significant *p*-value of that group for each biochemical measure.

### Variant annotations

We queried existing data and results from multiple resources to further annotate key variants and investigate the implicated loci. These additional datasets represent either the largest, most comprehensive, or most applicable dataset available to characterize the loci of interest for their associations with AD-related phenotypes, regulatory potential or other human diseases and phenotypes. Associations of GWS variant dosage with sqrt (CAA) were performed previously [37] and results were queried for key variants identified in this study. Cell-specific differential gene expression analysis was queried from Mathys et al. 2019 [71] between AD pathology and no pathology samples in six cell types (excitatory neurons, inhibitory neurons, microglia, oligodendrocytes, astrocytes, and oligodendrocyte precursor cells), downloaded from the supplemental material (Table S2) on June 10, 2019, and limited to genes ±1 Mb from the GWS variants in the Ensembl hg19 build (release 103) [72]. Only genes that passed study-level significance (FDR corrected *p*-value ≤0.01 and a fold change ≥0.25) in at least one cell type were included in our evaluation.

Summary statistics for the LOAD GWAS (Kunkle et al. 2019 [16]- NG00075, Lambert et al. 2013 [17]- NG00036) and CSF GWAS (Cruchaga et al. 2013 [73]- NG00049) were downloaded from NIAGADs. International Genomics of Alzheimer’s Project (IGAP) is a large three-stage study based upon genome-wide association studies (GWAS) on individuals of European ancestry. In stage 1, IGAP used genotyped and imputed data on 11,480,632 single nucleotide polymorphisms (SNPs) to meta-analyze GWAS datasets consisting of 21,982 Alzheimer’s disease cases and 41,944 cognitively normal controls from four consortia: The Alzheimer Disease Genetics Consortium (ADGC); The European Alzheimer’s disease Initiative (EADI); The Cohorts for Heart and Aging Research in Genomic Epidemiology Consortium (CHARGE); and The Genetic and Environmental Risk in AD Consortium Genetic and Environmental Risk in AD/Defining Genetic, Polygenic and Environmental Risk for Alzheimer’s Disease Consortium (GERAD/PERADES). In stage 2, 11,632 SNPs were genotyped and tested for association in an independent set of 8362 Alzheimer’s disease cases and 10,483 controls. Meta-analysis of variants selected for analysis in stage 3A (*n* = 11,666) or stage 3B (*n* = 30,511) samples brought the final sample to 35,274 clinical and autopsy-documented Alzheimer’s disease cases and 59,163 controls.

The 1000 genomes phase_3 (GBR) dataset was queried for variants in LD (± 50 kb, *r*^2^ ≥ 0.8, D′ ≥ 0.8) through Ensembl and NCBI LDlink (https://ldlink.nci.nih.gov/) [74]. The Genotype-Tissue Expression (GTEx) Project v8 (https://gtexportal.org/) [75, 76] was queried for significant eQTLs and sQTLs between August and December 2020.

A graphical description of the datasets integrated in each analysis is outlined in Fig. S7.

## Results

### Genome-wide association study identifies seven novel loci associated with AD brain biochemical endophenotypes

**Fig. 2:**
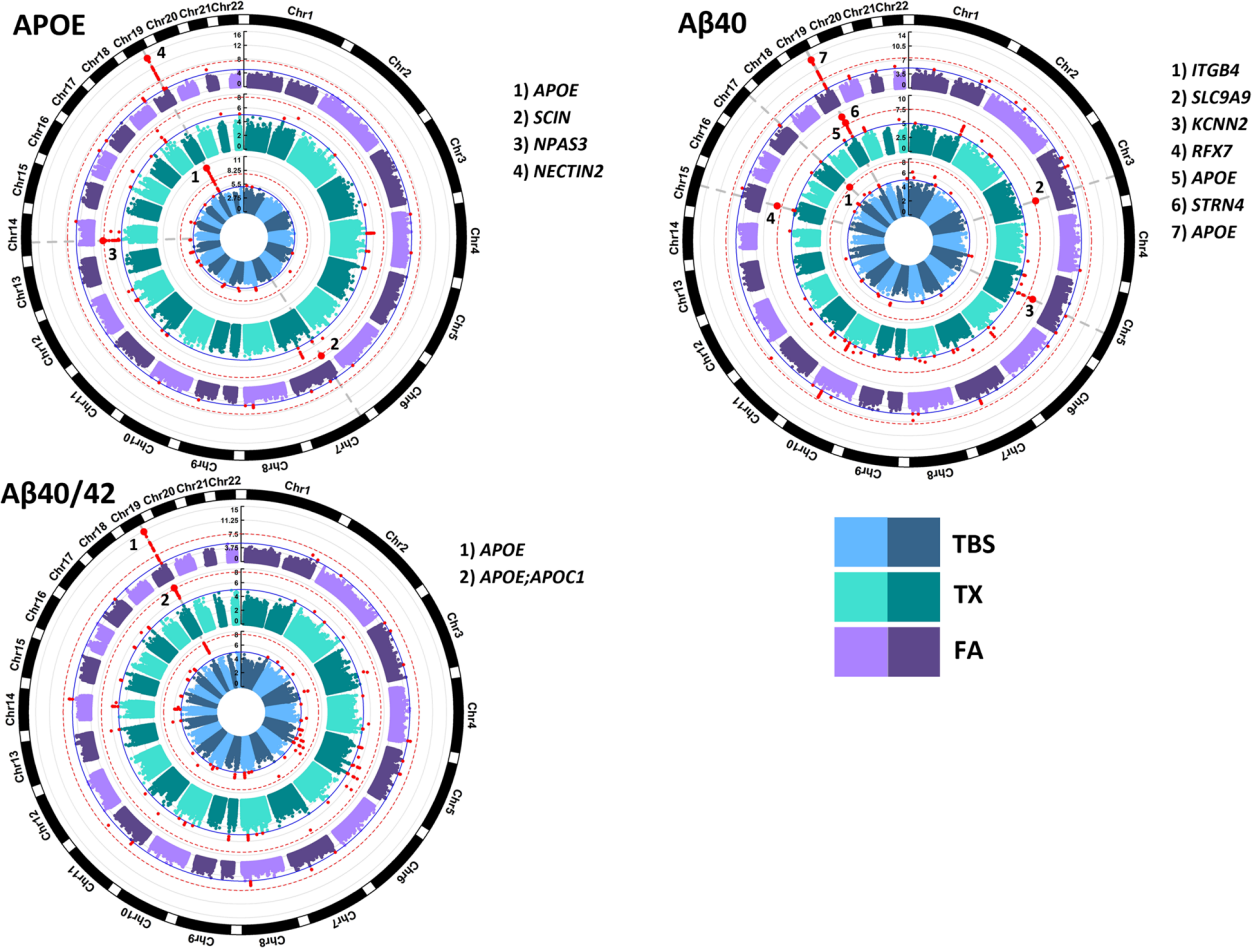
Circular Manhattan Plots of brain AD-related protein GWAS. Circular Manhattan plots for each protein measured in three biochemical fractions. Plots for proteins with SNPs that reach genome-wide significance (GWS) are shown. Red dotted line marks GWS threshold of *p*-value = 2.98E-08, solid blue line marks *p*-value = 1E-05. Top SNPs at GWS loci have dots increased in size and labeled with the closest gene name. SNPs with a *p*-value <1E-05 are colored red. Radial axes measure -log10(*P*-value). Inner most blue circle is the soluble TBS fraction, middle green circle is the membrane TX fraction and outer most purple circle is the insoluble FA fraction

**Fig. 3:**
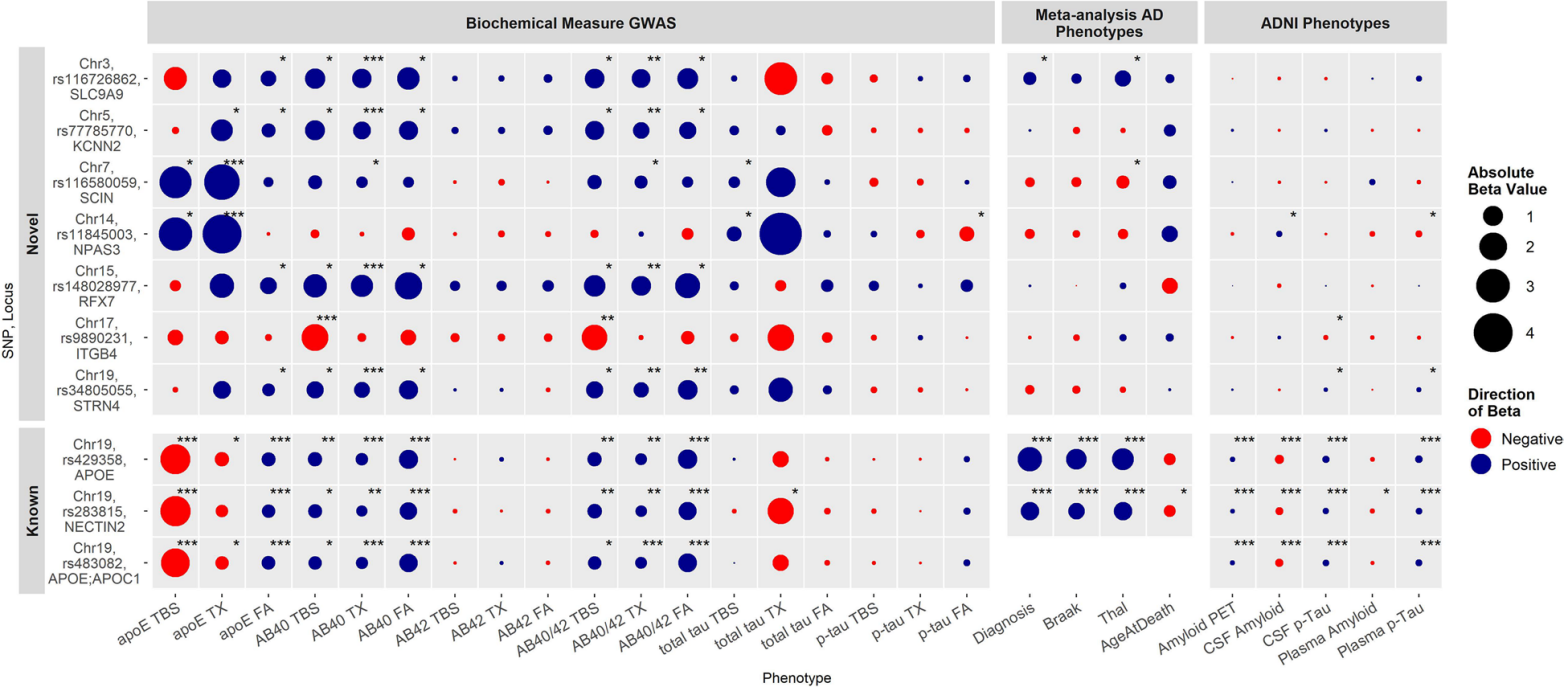
Association of Genome-wide significant SNPs across all Biochemical Measures, Meta-Analysis, and ADNI dataset with AD-related Phenotypes. Associations of novel (top) and known (bottom) GWS Index SNPs across all 18 biochemical measures in the Mayo Clinic Brain Bank cohort (*n* = 441) and results from meta-analysis with AD-related phenotypes in up to 4 cohorts (*n* = 3707). Meta-analysis was conducted using data from four independent autopsy datasets, namely the AMP-AD Mayo Clinic (*n* = 344), Mount Sinai Brain Bank (MSBB, *n* = 267), Rush (ROSMAP, *n* = 1091) and the Mayo expanded brain bank dataset (*n* = 2005). Mayo AMP-AD and expanded brain bank datasets were non-overlapping, and latter also included the 441 AD donors from the brain biochemical measures GWAS. Meta-analysis results are fixed effects models adjusted for sex and age at death when appropriate. Rs483082 was not significant after conditioning on rs429358 (*APOE*-ε4) and so was not carried forward for meta-analysis. Proxy SNPs genotypes were used for rs148028977 and rs116580059 in the Mayo expanded dataset. Note, Thal measures were only available from the expanded Mayo dataset and the AMP-AD Mayo dataset. Rs34805055 was not genotyped in the expanded Mayo dataset, therefore meta-analysis excluded this cohort for this SNP. ADNI associations include amyloid PET (*N* = 784), CSF amyloid (*N* = 1154), CSF p-Tau (*N* = 1151), plasma amyloid (*N* = 262), and plasma p-Tau (*N* = 787) adjusted for age and sex. Dot color indicates direction of beta value (blue = positive, red = negative), size of dot indicates absolute beta value. Associations with a *p*-value ≤2.98E-8 indicated by (***),1E-05 ≤ *p*-value < 2.98E-8 indicated by (**), and 0.05 ≤ *p*-value <1E-05 indicated by (*)

**Fig. 4:**
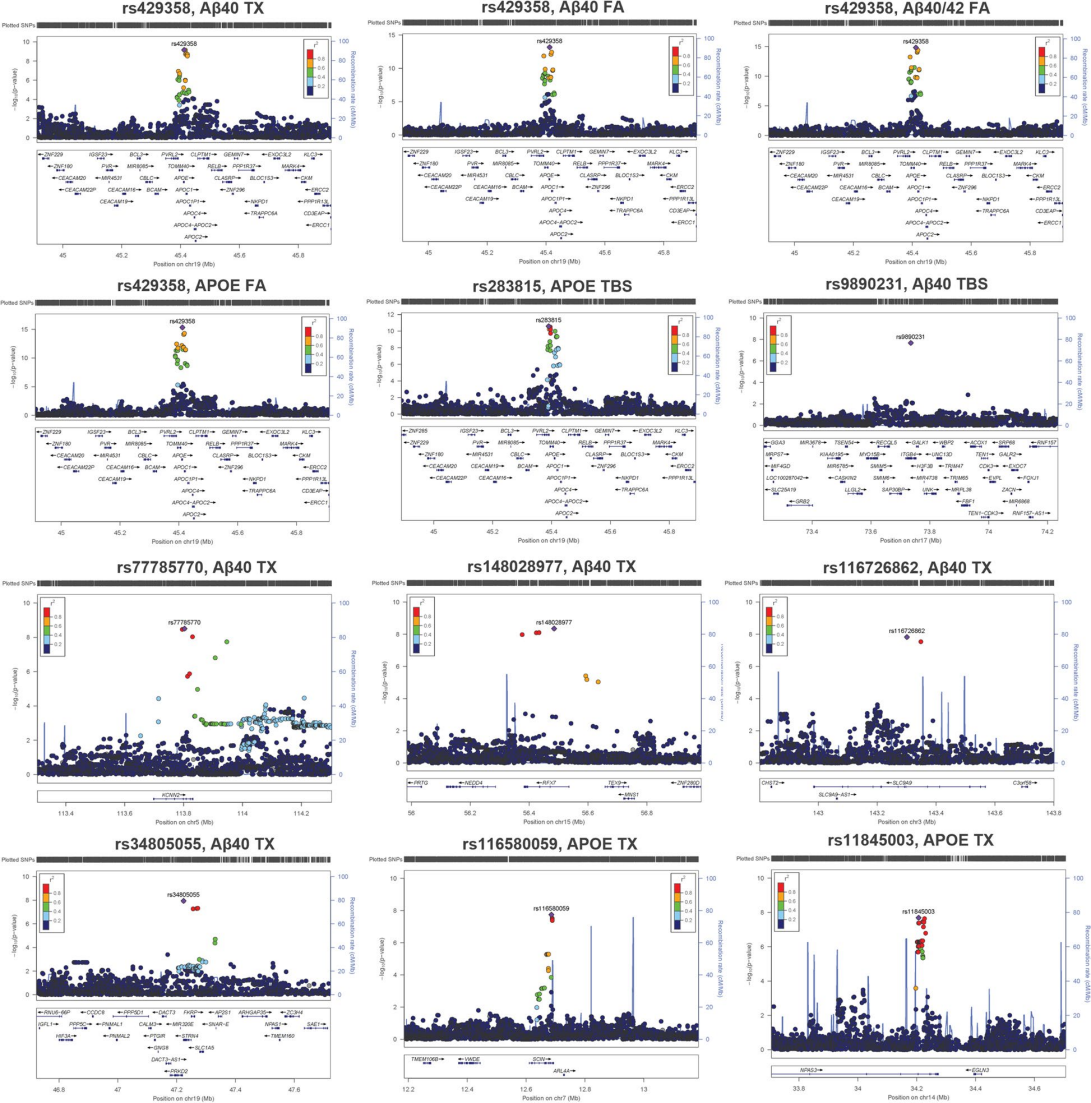
Locus Zoom Plots. Locus Zoom plots (locuszoom.org) of GWS SNPs showing associations +/− 500 kb from variant of interest (labeled). Right Y-axis shows the *p*-value, left Y-axis shows rate of recombination, and X- axis shows position on chromosome and nearby gene positions. Each plot point represents a variant in the dataset color coded by (*r*2) value

We utilized a cohort of 441 autopsy-confirmed AD cases from the Mayo Clinic Brain Bank with genome-wide genotypes and temporal cortex (TCX) biochemical measures of AD-related protein endophenotypes including APOE, Aβ40, Aβ42, total tau, and p-Tau from soluble (TBS), membrane (TX), and insoluble (FA) tissue fractions. Demographics including neuropathology scores are outlined in Table S1. Quantitative brain biochemical measures were previously collected and transformed to approximate a normal distribution (Table S2, Fig. S1) [32]. To identify genetic associations with brain levels of AD-related proteins, genome-wide association studies were performed for each normalized biochemical fraction as well as the normalized ratio of Aβ40/42, adjusting for age, sex, and the first three population principal components (PCs) in the primary model. When specified, we also adjusted for *APOE* genotypes as follows: conditional analysis on the *APOE*-ε4 tagging variant (rs429358) imputed dosages, including as covariates *APOE* diplotypes, the *APOE*-ε2 tagging variant (rs7412) dose, and/or *APOE*-ε4 dose, as well as stratified by *APOE* diplotype. Altogether, we identified genome-wide significant (GWS, *P* < 2.98 × 10^− 8^) SNP-endophenotype associations at 8 unique loci: 6 unique loci for Aβ40, 3 for APOE, and 1 for the ratio of Aβ40/Aβ42 (Table 1, Figs. 2, 3 and 4, Table S3).

**Table 1:** Description of genome wide significant SNPs

	Protein	Buffer	Transf	SNP	Chr	Locus	Position Annotation	Minor Allele	N	MAF	MAF Bin	HWE-P	Additive Model			Conditional ***APOE-ε***4 model			CADD Score	Regulome Rank
													Beta	(CI 95%)	P	Beta	(CI 95%)	P		
**Novel**	**Aβ40**	**TBS**	**ln**	**rs9890231**	**17**	***ITGB4***	**Intron** ***ITGB4***	**G**	**439**	**0.04**	**LF**	**1.00**	**−1.88**	**(−2.52–-1.23)**	**2.09E-08**	**−1.84**	**(−2.47–-1.21)**	**2.06E-08**	**3.371**	**5**
	**Aβ40**	**TX**	**ln**	**rs77785770**	**5**	***KCNN2***	**Intron** ***KCNN2***	**G**	**441**	**0.05**	**LF**	**0.60**	**0.79**	**(0.53–1.05)**	**3.12E-09**	**0.72**	**(0.47–0.97)**	**2.15E-08**	**0.977**	**5**
	**Aβ40**	**TX**	**ln**	**rs148028977**	**15**	***RFX7***	**Intron** ***RFX7***	**T**	**441**	**0.02**	**LF**	**1.00**	**1.24**	**(0.83–1.64)**	**4.47E-09**	**1.1**	**(0.71–1.49)**	**6.98E-08**	**12.64**	**5**
	**Aβ40**	**TX**	**ln**	**rs116726862**	**3**	***SLC9A9***	**Intron** ***SLC9A9***	**T**	**441**	**0.03**	**LF**	**1.00**	**0.95**	**(0.63–1.27)**	**1.51E-08**	**0.57**	**(0.38–0.77)**	**2.78E-08**	**3.507**	**3a**
	**Aβ40**	**TX**	**ln**	**rs34805055**	**19**	***STRN4***	**Intron** ***STRN4***	**T**	**441**	**0.08**	**MF**	**1.00**	**0.61**	**(0.41–0.82)**	**1.15E-08**	**0.85**	**(0.54–1.16)**	**1.53E-07**	**6.379**	**4**
	**APOE**	**TX**	**sqrt**	**rs116580059**	**7**	***SCIN***	**Intron** ***SCIN***	**C**	**439**	**0.03**	**LF**	**1.00**	**3.34**	**(2.20–4.48)**	**1.82E-08**	**3.38**	**(2.24–4.51)**	**1.08E-08**	**1.254**	**3a**
	**APOE**	**TX**	**sqrt**	**rs11845003**	**14**	***NPAS3***	**Intron** ***NPAS3***	**T**	**439**	**0.02**	**LF**	**1.00**	**4.05**	**(2.66–5.44)**	**2.05E-08**	**4.04**	**(2.65–5.42)**	**1.99E-08**	**7.418**	**4**
**Known**	**Aβ40**	**TX**	**ln**	**rs429358**	**19**	***APOE***	**Exon** ***APOE***	**C**	**441**	**0.4**	**HF**	**0.07**	**0.37**	**(0.25–0.48)**	**6.88E-10**	**–**	**–**	**–**	**0.007**	**4**
	**Aβ40**	**FA**	**ln**	**rs429358**	**19**	***APOE***	**Exon** ***APOE***	**C**	**441**	**0.4**	**HF**	**0.07**	**0.91**	**(0.68–1.15)**	**6.75E-14**	**–**	**–**	**–**	**0.007**	**4**
	**Aβ40/42**	**TX**	**ln**	**rs483082**	**19**	***APOE/APOC1***	**Intergenic**	**T**	**441**	**0.42**	**HF**	**0.49**	**0.33**	**(0.22–0.44)**	**2.11E-08**	**0.29**	**(−0.08–0.66)**	**0.12**	**10.25**	**4**
	**Aβ40/42**	**FA**	**ln**	**rs429358**	**19**	***APOE***	**Exon** ***APOE***	**C**	**441**	**0.4**	**HF**	**0.07**	**0.94**	**(0.72–1.16)**	**1.48E-15**	**–**	**–**	**–**	**0.007**	**4**
	**APOE**	**FA**	**ln**	**rs429358**	**19**	***APOE***	**Exon** ***APOE***	**C**	**441**	**0.4**	**HF**	**0.07**	**0.49**	**(0.38–0.60)**	**4.53E-16**	**–**	**–**	**–**	**0.007**	**4**
	**APOE**	**TBS**	**sqrt**	**rs283815**	**19**	***APOE/NECTIN2***	**Intron** ***NECTIN2***	**G**	**441**	**0.42**	**HF**	**0.02**	**−2.41**	**(−3.10–-1.72)**	**2.60E-11**	**−1.51**	**(−2.74–-0.27)**	**1.75E-02**	**1.827**	**7**

Descriptions of top genome-wide significant (GWS) SNPs associated with each biochemical measure in N = 441 AD cases. Seven are novel and three are known. Models include an additive model adjusted for age, sex, and PC1-3, and a conditional model conditioning on APOE-ε4 (rs429358) adjusted for age, sex, and PC1-3

*Transf* Transformation, *N* Number, *MAF* Minor Allele Frequency, *HWE-P* Hardy Weinberg Equilibrium *P*-value, *CI* Confidence Interval, *P* P-value

Seven of these loci involve novel intronic variants that have not been previously implicated in genetic association studies of AD or related endophenotypes: rs116580059 near *SCIN* for APOE in TX fraction (rs116580059-APOE_TX_ (*SCIN*)), rs11845003-APOE_TX_ (*NPAS3*), rs116726862-Aβ40_TX_ (*SLC9A9*), rs148028977-Aβ40_TX_ (*RFX7*), rs34805055-Aβ40_TX_ (*STRN4*), rs77785770-Aβ40_TX_ (*KCNN2*) and rs9890231-Aβ40_TBS_ (*ITGB4*). Assessment of the index SNPs at each locus across all biochemical measures determined that each is nominally (*P* < 0.05) associated with additional biochemical measures (Fig. 3). The estimated proportion of biochemical measure variance explained by the index SNPs based on the *R*^2^ of linear regression models ranged from 7 to 27%, with *APOE-ε*4 (rs429358) alone explaining between 6.6 to 14% (Table S4).

More broadly, we detected 1813 variants with a *P* < 1 × 10^− 5^ ranging from 26 variants for total tau_TBS_ to 341 for APOE_TX_ (Table S3). While not reaching GWS, the most significant SNPs for the remaining traits include, Aβ42: TBS-rs147370282, TX-rs10219590, FA-rs461939; Aβ40/42: TBS-rs9890231, TX-rs483082; total tau: TBS-rs34678552, TX-rs76878089, FA-rs1634993; and p-Tau: TBS-rs2294557, TX-rs10987782, and FA-rs11651012 (Fig. S2, Table S3). Interestingly we found associations that approach GWS for total tau_TBS_ (*P* = 8.03E-07) and total tau_TX_ (*P* = 9.48E-06) with rs117691004 which is located in the *PRKN* gene known to play a role in Parkinson’s Disease [77] (Table S3).

### Multiple variants at the *APOE* locus associate with brain biochemical measures of AD-related proteins

**Fig. 5:**
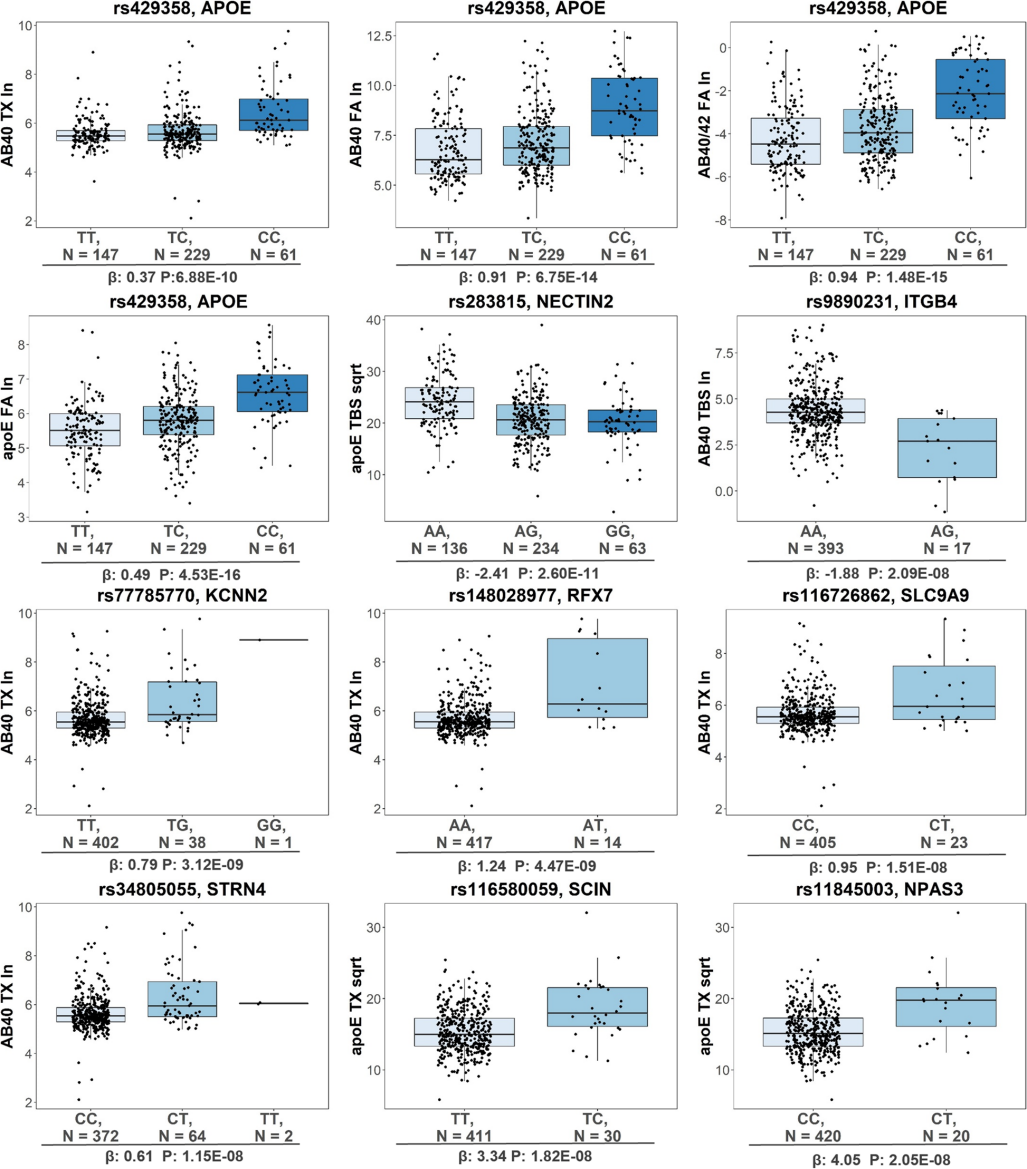
Box Plots of Genome-wide Significant SNP Genotypes. Box plots of hard-call genotypes for each genome-wide significant SNP from each biochemical measure GWAS. Each dot represents an individual sample, N = Number. Variant rs number and gene closest to GWS SNP listed at the top, beta and *p*-value listed at the bottom, biochemical measures on the y-axis. Genotype: Light blue- Homozygous major, Medium blue- Heterozygotes, Dark blue- Homozygous minors

Presence of the *APOE-*ε4 allele has previously been reported to associate with biochemical measure levels in this dataset [32], however, using the GWAS data we can explore the effects of *APOE*ε4 dose and additional genetic variation at this locus. We found a total of 30 unique GWS variants at or proximal to the *APOE* gene with at least one associated with six of the biochemical measures (Table S3). The known exonic AD risk *APOE-ε*4 tagging variant (rs429358) was the most significant SNP for four of the traits: Aβ40_TX_, Aβ40_FA_, Aβ40/42_FA_, APOE_FA_. The proximal *NECTIN2* intronic variant (rs283815) was the top SNP for APOE_TBS_ (Figs. 4 and 5), and an intergenic SNP between *APOE* and *APOC1* (rs483082) was the most significant for Aβ40/42_TX_. These two SNPs are in linkage disequilibrium (LD) with *APOE-ε*4 in our dataset (rs283815: *r*^2^ = 0.73, D′ = 0.87; rs483082: *r*^2^ = 0.92, D′ = 0.99) which is likewise associated with the same biochemical traits (Fig. 3, Table S3). In an *APOE-ε*4 conditional analysis, only rs283815 remains nominally significant (Table 1). We further examined the rs283815-APOE_TBS_ association after adjusting for *APOE-*ε2 and *APOE-*ε4 dose, for *APOE* diplotypes, and in the *APOE*-33 only sample subset (*N* = 141, β = − 3.79, *P =* 2.16E-03) finding that this association remained nominally significant (Table S5). Taken together, this suggests that the effects of rs483082 and *APOE-*ε4 on Aβ40/42_TX_ levels likely represent the same signal. However, because the rs283815-APOE_TBS_ association is present even in the *APOE*-33 only sample subset, as well as after adjusting for combinations of *APOE* diplotypes, our results suggest that more than one genetic variant at the *APOE* locus beyond that of the *APOE-*ε4 signal likely contribute to soluble APOE levels in the TCX. The rs283815 variant has previously been implicated in AD risk in males [78] and imaging of cerebral amyloid deposition [79], although it did not survive adjustment for *APOE-ε*4 in the latter. These variants at the *APOE* region (rs429358, rs283815, and rs483082) represent the most significant associations across all fractions of Aβ40, Aβ40/42, and APOE, but no fractions of Aβ42, total tau, or p-Tau (*P* > 0.031) (Table S6); indicating that they likely impact disease risk through effects on APOE and Aβ40, but not tau. Furthermore, the direction of association for APOE fractions indicates a shift in biochemical state with minor allele carriers having lower soluble APOE (APOE_TBS_) and higher insoluble APOE (APOE_FA_) (Fig. 3), suggesting a role in promoting aggregation of APOE rather than overall levels.

### AD brain biochemical endophenotype GWS variants also associate with disease risk, age at death, and AD-related neuropathology and biomarkers

To further characterize the GWS variants with respect to other AD-related phenotypes, we evaluated their association with AD risk, AD-related neuropathological variables (Braak stage, Thal phase, cerebral amyloid angiopathy = CAA), AD-related biomarkers (amyloid [^18^F] Florbetapir PET scans, plasma total Aβ42, and phosphorylated tau (pTau), CSF Aβ42, total tau (t-tau) and pTau) and age at death in additional samples.

We expanded the cohort size and validated the GWAS genotypes by genotyping these variants or their proxies in the GWAS study samples (*N* = 441) and additional Mayo Clinic Brain Bank (MCBB) participants (*N* = 1564) using TaqMan assays or Sanger sequencing (Table S7). We refer to these 2005 participants as the Mayo Clinic Expanded Cohort. A high level of concordance, > 98%, was observed between the array-based genotyped or imputed alleles and those collected by TaqMan and sequencing (Table S8). Association results using the TaqMan genotypes in place of the array-based genotypes showed only minor variations in effect size and significance, demonstrating consistency of the results (Table S5). Genotypes for the index GWS variants were also extracted from three independent whole genome sequence (WGS) datasets available from the Accelerating Medicines Partnership AD (AMP-AD) study through the AD knowledge portal (www.synapse.org) which includes the Mayo Clinic RNAseq (Mayo, *n* = 344) [42], Mount Sinai Brain Bank (MSBB, *n* = 267) [43], and Rush Religious Orders Study and Memory and Aging Project (ROS-MAP, *n* = 1091) [44] studies (Table S7). We note that the AMP-AD Mayo and Mayo Clinic Expanded Cohort are non-overlapping. Meta-analyses for available genotypes and common AD-related phenotypes were conducted across these 4 independent datasets for each SNP using fixed and random effects models (Fig. 3, Fig. S3, Table S9).

In addition, we investigated the association of GWS variants with other AD-related endophenotypes that were not available in the aforementioned datasets. We queried a previous GWAS of CAA [37] to determine the association of the GWS variants in the current study with this vascular AD pathology. We also evaluated the association of the GWS variants with AD-related biomarkers including amyloid PET measures, CSF and plasma Aβ and tau in the ADNI dataset.

As expected, the *APOE-ε*4 variant (rs429358) and the proximal *NECTIN2* variant (rs283815) significantly associated with AD risk, Thal phase, Braak stage, amyloid PET, CSF amyloid and p-Tau, plasma p-Tau and as reported previously for *APOE-ε*4, also CAA [32]. Rs283815 was also associated with age at death and plasma amyloid, although the rs283815 associations were no longer significant after adjustment for *APOE-*ε2 or *APOE-*ε4 (Fig. 3, Tables S9 and S10). Of the novel variants, we found rs116726862 (*SLC9A9* intron, increased Aβ40_TX_) was associated with higher Thal phase (*P =* 7.50E-03), increased CAA (*P* = 4.70E-02), and a trend for increased AD risk (*P =* 0.07), higher Braak stage (*P =* 0.07), and higher plasma p-Tau levels (*P* = 0.08). The *SCIN* intronic SNP rs116580059 (increased APOE _TX_) was associated with lower Thal phase (*P* = 0.015) and a trend (*P* = 0.06) for lower Braak stage. We found rs11845003 (*NPAS3* intron, increased APOE_TX_) associated with increased CSF amyloid levels (*P* = 0.02), decreased plasma p-Tau levels (*P* = 0.04), and trends for decreased amyloid PET (*P* = 0.09). Rs9890231 (intron *ITGB4*, decreased Aβ40_TBS_) associated with decreased CSF p-Tau (*P* = 0.01) and a trend for lower amyloid PET (*P* = 0.07). The intronic *STRN4* variant rs34805055 (increased Aβ40_TX_) associated with increased CSF p-Tau (*P* = 0.03) and plasma tau levels (*P* = 0.04) (Fig. 3, Tables S9 and S10).

In all cases, we find these additional AD-related phenotype associations are entirely consistent with what we would expect based on prior knowledge. Specifically, higher amyloid PET, CSF pTau, plasma pTau, lower CSF and plasma Aβ have congruent associations with the AD risk variant at the *APOE* locus and other variants that behave like *APOE* with respect to brain biochemistry associations (i.e. *SLC9A9, STRN4* variants). In contrast, variants that have a pattern of brain biochemistry associations opposite to that of *APOE* also have opposite direction of associations for the other AD-phenotypes as well (i.e *NPAS3* and *ITGB4* variants). Although not all associations would survive Bonferroni correction, these biologically congruent associations facilitate identification of potential molecular mechanisms connecting genetic variants, biochemical measures, and other AD phenotypes. Taken together, these results validate the array-based genotype calls by an independent assay. Importantly, they also implicate at least two of the novel variants more broadly in AD risk and neuropathology (variants near *SLC9A9* and *SCIN*), and four with additional AD-related endophenotypes (near *SLC9A9*, *NPAS3*, *ITGB4,* and *STRN4*) in a direction that is consistent with the brain biochemical findings and known associations with the well-established *APOE* risk variant.

### Brain transcriptome analyses implicate expression dysregulation at some of the novel AD brain biochemical endophenotype loci

We hypothesized that some of the variants might function through their influence on the expression or splicing of nearby genes. We performed *cis*-expression quantitative trait locus (*cis*-eQTL) analysis (SNP ± 1 Mb) in three AMP-AD transcriptome datasets collected from seven brain regions of AD cases and controls available through the AD knowledge portal and also queried independent results from the GTEx portal (www.gtexportal.org/home/, queried 08/2020) [75, 76]. We found that rs34805055-Aβ40_TX_ (intron *STRN4*) was significantly associated with the downregulation of *PRKD2* gene expression in the Mayo Clinic TCX dataset (β = − 0.29, *q-value* = 2.6E-03) and *RN7SL364P* in the ROS-MAP dorsolateral prefrontal cortex (DLPFC) dataset (β = − 0.38, *q-value* = 3.0E-02). The *PRKD2* gene is located approximately 3 kb downstream of rs34805055 while *RN7SL364P* is a pseudogene located within an intron of *PRKD2*. In the GTEx dataset, *PRKD2* expression and splicing QTLs were found for this variant in healthy brain cortex tissue (Normalized Effect Size (NES) = − 0.46, *P* = 4.1E-05), and other tissue types. The rs283815-APOE_TBS_ (*NECTIN2* intron) variant significantly associated with *TOMM40* splicing in healthy cerebellum tissue (NES = − 0.94, *P* = 3.80E-16), whilst rs9890231-Aβ40_TBS_ (intron *ITGB4*) associated with *ITGB4* splicing in several tissues including healthy tibial nerve tissue (NES = − 1, *P* = 6.50-09). Altogether, these results indicate that the rs34805055-Aβ40_TX_ variant may influence gene regulation and splicing of the *PRKD2* gene rather than the index gene *STRN4*, while rs9890231-Aβ40_TBS_ and rs283815-APOE_TBS_ may influence splicing of *ITGB4* and *TOMM40*, respectively_._ The remaining novel index variants do not appear to influence gene regulation in the CNS at the bulk tissue level.

We next investigated the colocalization of the significant GWAS and QTL signals at each locus by testing for colocalization under single and multiple causal variant assumptions. We found that none of the traits (GWAS and QTL pairs) showed evidence of colocalization under the assumption that a locus has a single causal variant with both traits. Relaxing this assumption to allow for multiple causal variants at a locus to associate with a trait, we found the rs34805055-Aβ40_TX_ (*STRN4*) trait and Mayo TCX *PRKD2* eQTL showed evidence of colocalization (H4 posterior probability = 0.999). It should be noted, however, there was only one credible set identified in this analysis with rs34805055 and rs62134781 being the top signals in the GWAS and eQTL datasets, respectively. A single credible set suggests a single casual variant at the locus for both traits, however, our original analysis under this assumption did not show evidence of colocalization and these two variants are not in LD (*R*^2^ = 0.1347, D′ = 1). No other traits showed colocalization with multiple causal variants.

We also examined each implicated locus (variant ±1 Mb, hg19) to determine if there were differentially expressed (DE) genes between AD cases and controls. We used the AMP-AD RNAseq datasets [42–44] to assess 267 expressed genes. We found that while all loci harbored DE genes in at least 1 brain region, 3 index genes and 1 gene implicated through the above QTL analysis were consistently DE in 2 or more brain regions. *KCNN2* was downregulated in AD for 3 datasets while *RFX7, SLC9A9,* and *PRKD2* were upregulated in AD for 2 (Table S11). Bulk tissue profiling captures changes across multiple cell types and may miss cell-specific molecular changes so we also queried results from a published single-cell RNAseq (scRNA-seq) dataset [71]. We found 67 genes at the implicated loci that were DE in at least one cell type between samples with AD pathology and those with no AD pathology, 90% of which were dysregulated in neurons (Table S12). Of the index genes, *ITGB4* was upregulated in astrocytes, *NECTIN2* was downregulated in neurons, while *APOE* was upregulated in neurons and microglia, and downregulated in astrocytes in AD. Interestingly, although we focus on the AD pathology vs no pathology analyses, Mathys et al. [71] also reported DE genes between early and late AD pathology in which we see upregulation of *PRKD2* in neurons (fold change = 0.51) in late AD. Collectively these results implicate dysregulation of *PRKD2* and *ITGB4* at the novel loci identified by this study and *APOE*, *NECTIN2* and *TOMM40* at the established Chr19q13 AD locus (Fig. S4). Larger datasets with genetic and single-cell data will be needed to further explore whether these variants influence cell-specific gene expression changes.

### Genes at the AD brain biochemical endophenotype GWAS loci are implicated in neuronal health and disease

We hypothesized that variants either in LD with the GWS index variants or others at the AD brain biochemical endophenotype GWAS loci may also associate with function or disease(s) of the central nervous system (CNS).

First, we identified variants in LD with the GWS index variants by querying the AMP-AD WGS Mayo TCX and CER datasets as well as the 1000 Genomes GBR dataset. We then searched these LD variants in the GTEx database as well as the GWAS catalog for significant associations beyond those already reported for the index variants (queried 09/28/2022). Eight of the nine GWS index variants had variants in LD with them. All but one of these were in noncoding regions, the exception being rs157581 in LD with rs283815, a synonymous missense variant in *TOMM40* (Table S9). At this same locus, we found multiple additional variants in LD with rs283815 at *NECTIN2*-APOE_TBS_ locus with previous associations to AD, CAA, cognition, and AD biomarkers including CSF levels of Aβ42 and tau [78–84]. Interestingly, we also identified a complete proxy of the index variant rs116726862 at *SLC9A9*-Aβ40_TX_ locus, rs115134872 (D′ = 1, *r*^2^ = 1), previously associated with survival in amyotrophic lateral sclerosis [85].

Next, we investigated all variants present in the index genes through a gene search in the GWAS catalog (https://www.ebi.ac.uk/gwas/, queried 06/07/2021) [86]. Five of the seven novel index genes have variants that associate (*P* < 1E-05) with AD-related phenotypes (Table S9). These include the *SLC9A9* locus associated with brain Aβ40_TX_ (i.e., *SLC9A9*-Aβ40_TX_ locus) in this study and working memory [87], response to cholinesterase inhibitors in AD [88] and epistatic interactions with tau measurements [89]. *SLC9A9*-Aβ40_TX_ locus was also implicated in the neuropsychiatric disorders of autism [90, 91] and attention deficit hyperactivity disorder [92].

Another brain biochemical endophenotype GWAS locus with AD and other neuropsychiatric disease-related associations was *NPAS3*-APOE_TX_ which is also associated with neuritic and diffuse plaque measurements [93], CSF levels of soluble TREM2 [94], epistatic interactions with tau measurements [89], schizophrenia [95, 96] and bipolar [95, 97, 98] disorder. The *KCNN2*-Aβ40_TX_ locus was also associated with age of onset for AD [99], epistatic interactions with amyloid [89], schizophrenia [95, 96], bipolar [95, 97, 98] disorder and hippocampal sclerosis [100]. Additionally, *RFX7*-Aβ40_TX_ and *SCIN*-APOE_TX_ loci have associations with regional brain volume [101] and inflammation markers [102], respectively.

This convergent evidence supports important roles for most of the AD brain biochemical endophenotype GWAS genes and loci in neuronal health and disease.

### Gene set enrichment analysis identifies shared and distinct pathways among GWAS genes for different AD brain biochemical measures

**Fig. 6:**
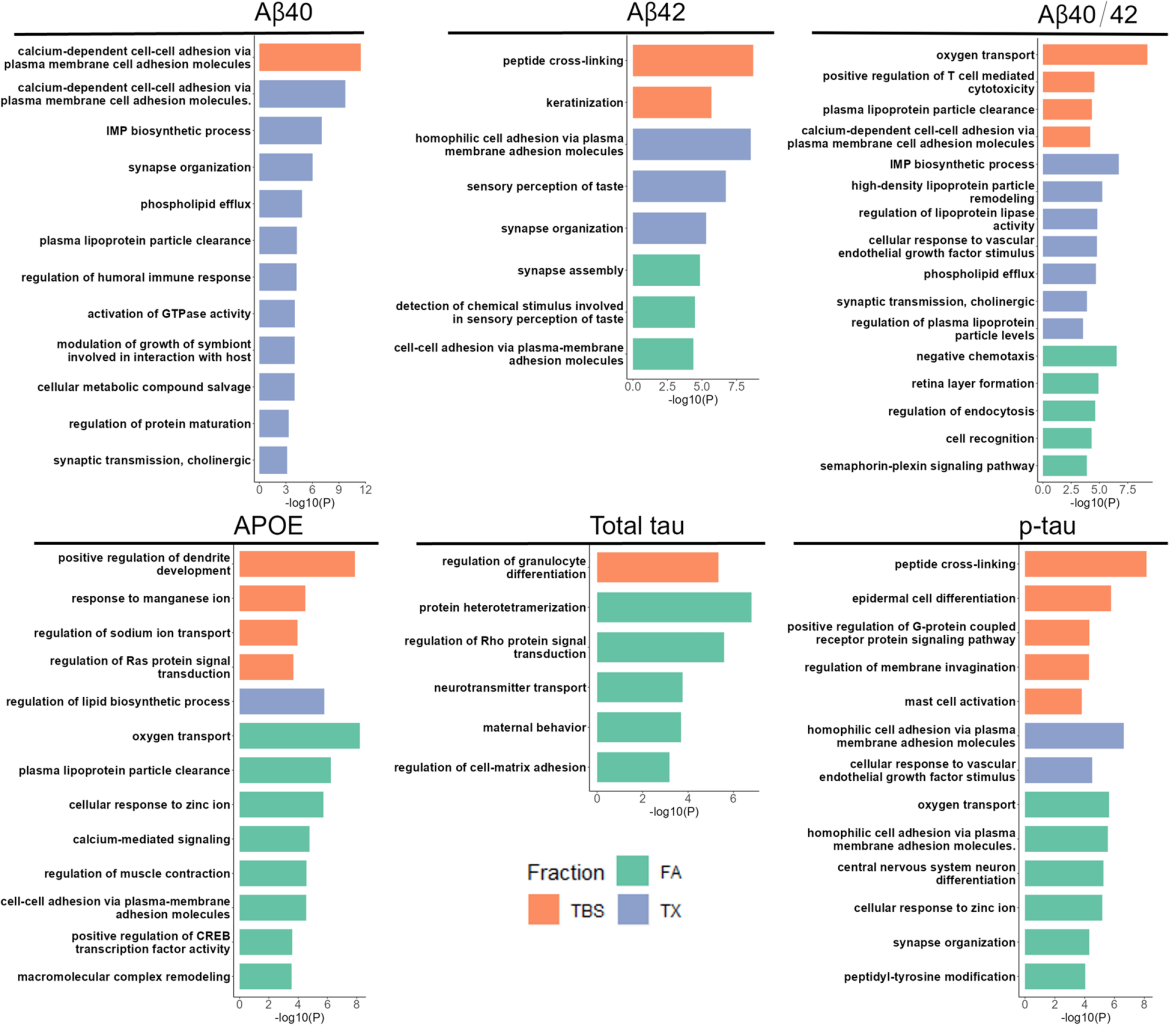
Gene set enrichment analysis in all biochemical measures. Gene set enrichment was performed via GSA-SNP2 with the MiSigDB (c5.all.v5.2). Significantly enriched pathways (q-value < 0.05) were matched with Gene Ontology (GO) IDs and input in REViGO for reduction of redundant pathways and summarization. Significant pathway groups from REViGO and the highest *p*-value of that group are plotted in bar plots for each protein. X-axis = −log10(*P*-value), Orange = soluble (TBS) fraction, Blue = membrane (TX) fraction, Green = insoluble (FA) fraction

To identify pathways that are enriched for each AD brain biochemical fraction GWAS we performed gene set enrichment analysis using GSA-SNP2 [65] with Gene Ontology (GO) terms [65]. We identified both shared and distinct significantly enriched pathway terms for each biochemical measure GWAS (Fig. 6). Shared biological pathway terms have known roles in AD such as synapse organization, cell-to-cell adhesion, and immune-related processes. Distinct enrichment for different biochemical fractions (TBS, TX, FA) was related to known functions for each protein, indicating that discrete molecular mechanisms likely influence specific biochemical states of each AD-related protein. For example, APOE is known to function in lipid metabolism and we found enrichment for lipoprotein clearance pathways. Notably, for soluble Aβ42_TBS_ and p-Tau_TBS_ we found enrichment in peptide cross-linking pathways indicating a genetic influence on systems that may play a role in the transition from soluble to aggregated forms of these proteins. Further, we see the enrichment of synapse organization and central nervous system neuron differentiation pathways for p-Tau_FA_. This is consistent with the well-established knowledge that NFTs comprising hyper-phosphorylated tau correlate with neuronal loss and severity in AD [103]. Interestingly, we found distinct enrichment in endocytosis regulation pathways for Aβ40/42_FA_ and sensory perception of taste pathways for Aβ42_TX_ and Aβ42_FA_. These results implicate variants proximal to genes involved in both known and novel pathways that may play a role in AD pathogenesis through impacts on specific or multiple AD-related proteins and their distinct biochemical states in the brain.

### Some AD risk GWAS variants also influence distinct biochemical measures

Finally, we wanted to determine if there are shared genetic risk factors for AD risk and specific brain AD-related biochemical measures. We queried large-scale AD risk GWAS [16, 17] for the novel variants identified in this study but did not find a significant association (*P* > 0.05) for those outside the *APOE* locus. This would suggest that while the GWS variants may influence these brain biochemical endophenotypes, they do not have a statistically significant impact on the more heterogenous phenotype of AD risk. We also investigated 28 GWS late-onset AD (LOAD) established risk variants for association with each biochemical measure [16]. Of the 26 variants present in our dataset, only associations with the *APOE*ε4 tagging variant survived Bonferroni correction for 26 tests (*P* < 1.93E-03), however; 9 others were nominally significant (*P* < 0.05) for at least one biochemical fraction with six having directions of effect consistent with AD risk, based on prior knowledge, and the direction of effect for the *APOE*ε4 tagging variant (Table S13). These include *APOE-ε*4-rs429358, discussed previously, and the risk allele for rs9331896 (C) at the *CLU* locus which associates with higher brain APOE_TBS_, APOE_TX_ and total tau_TBS_, and lower p-Tau_TX_. The risk allele for rs73223431 (T) at the *PTK2B* locus associates with increased p-Tau_TX_ and p-Tau_FA_, but not APOE or Aβ measures. The protective allele of rs1080826 (A) at the *EPHA1* locus associated with lower levels of Aβ42_TX_, Aβ42_FA_ and total tau_FA_ (Table S13). While associations outside the *APOE* locus would not survive Bonferroni correction, these results using unique biochemical measures from brain tissue can still indicate the underlying pathological mechanisms by which these established AD-risk variants might influence disease.

## Discussion

Genetic, model system, and neuropathology studies have clearly established Aβ, tau and APOE as disease hallmarks, biomarkers and therapeutic targets in AD and other neurodegenerative diseases [104–106]. Even though there are several strategies targeting these molecules for therapeutic benefit in AD, critical knowledge gaps hinder progress. All three molecules undergo complex processing and exist in heterogeneous biochemical states in the human brain. Discovering genetic and other factors contributing to this molecular complexity and biochemical heterogeneity can yield novel therapeutic avenues. Further, the relationship of the various biochemical states of these molecules and their genetic determinants with other AD-related outcomes can help clarify the beneficial vs. detrimental mechanism of action for targeted therapies. Finally, a comprehensive genetic map of the various biochemical states of key AD proteins can help pave the way for personalized medicine targeting specific perturbed pathways in an individualized fashion.

In this study we sought to identify the genetic determinants contributing to variability in the brain biochemical states of key AD-related proteins. We report the identification of eight independent GWS loci that associate with brain levels of five hallmark AD-related proteins isolated from three tissue fractions. Seven loci are novel and associated with Aβ40, APOE, and Aβ40/Aβ42 biochemical levels. Aside from these novel loci, we also observe significant associations across the biochemical measures for the *APOE-*ε4 tagging variant (rs429358). Notably, we detected a signal within *NECTIN2* nearby the *APOE* locus that cannot be entirely explained by *APOE-ε*4. These results demonstrate the contribution of genetic factors besides *APOE-ε*4 at the *APOE* locus to the variability of brain biochemical states of AD-related proteins.

Our study also provides insights into the pathological mechanisms through which the *APOE* and novel loci may act through to influence the brain biochemical measures. Although the *APOE-ε*4 variant (rs429358) and the *NECTIN2* variant (rs283815) were associated with measures of APOE, Aβ40, and Aβ40/42 ratio, these variants did not show significant associations with Aβ42, total tau, and p-Tau, consistent with previous findings in this dataset [32]. These results suggest that the *APOE* locus may influence mechanisms associated with levels of APOE and Aβ40 proteins rather than Aβ42 or tau in the brain. This contrasts with some previous studies that suggest the *APOE* locus does affect tau levels independent of Aβ [73, 107, 108], others showing brain region specific effects of *APOE-ε*4 [109–111], while another study showed an interaction effect [112]. In our current study, while we cannot rule out the possibility there may be associations at the *APOE* locus that affect Aβ42 or tau brain biochemical levels, based on the 95% confidence intervals of these associations (Table S6), these effects would likely be small. Nonetheless, it will be important for future studies to investigate this further in additional datasets, particularly those with tissue fraction specific data such as ours, case-control cohorts, and longitudinally.

Importantly, this study identified additional, biologically congruent associations for five of the seven novel loci including *SLC9A9*, *SCIN, NPAS3, ITGB4,* and *STRN4*. Based on the associations of the *APOE* locus, we would expect increased Aβ40_TX_ (*SLC9A9* and *STRN4*) to correlate with increased AD risk while increases in APOE_TX_ (*SCIN* and *NPAS3*) and Aβ40_TBS_ (*ITGB4*) would correlate with decreased AD risk. Remarkably, we see these relationships recapitulated in the independent datasets we evaluated. The likely detrimental variants at loci *SLC9A9* and *STRN4* associate with increased AD risk, Braak, Thal, CSF p-Tau, and/or plasma p-Tau levels, while the likely beneficial variants at loci *SCIN, NPAS3,* and *ITGB4* associate with decreased Thal and CSF p-Tau levels, increased CSF amyloid levels, and/or trending with decreased amyloid PET measures. These biologically congruent associations in independent datasets provide support that the effects of these novel loci on brain biochemical levels may have roles in the broader pathophysiology of AD.

Of the novel loci, *SLC9A9* which significantly associates with Aβ40_TX_ also has nominal associations and trends with higher levels of CAA, Braak, Thal, plasma p-Tau, and AD risk. In a previous study, Aβ40_TX_ levels were shown to positively correlate with CAA scores [32]. Our findings suggest that the *SLC9A9* locus may influence AD neuropathologies, including CAA, by mediating brain Aβ40_TX_ levels. *SLC9A9* encodes a sodium hydrogen exchanger with multiple functions in regulating the endosome, an organelle critical for the processing of amyloid [113]. Notably, the *SLC9A9* locus has associations with other AD-related phenotypes such as working memory [87], response to cholinesterase inhibitors in AD [88], and other neuropsychiatric diseases [114–116]. Based on our findings, we postulate that fundamental functions of SLC9A9 in the endosome, including amyloid processing, may underlie its influence on AD and other neuropsychiatric disease-related outcomes.

Many of the other index genes discovered in our brain biochemical endophenotype GWAS have established roles relevant to AD pathology, neurological disorders, and brain function. The *KCNN2*, *RFX7*, *STRN4* and *ITGB4* loci significantly associate with brain Aβ40 levels, the first three for the membrane-bound (TX) and the last for the soluble (TBS) fraction. *KCNN2,* which encodes a calcium-activated potassium channel, resides at a locus with many other AD-related and neuropsychiatric associations (Table S9). In a transcriptional network analysis, *KCNN2* was the top-ranked network driver gene for classifying AD cases vs. controls [117] and has been shown to have alternative splicing in AD [118]. *STRN4*, like *SLC9A9* and *KCNN2*, encodes a membrane-bound protein. STRN4 was reported to be a key binding partner and possible regulator of MAP4K [119], the inhibition of which was shown to be neuroprotective [120, 121]. MAP4K is an upstream regulator of YAP [119], the deficiency of which by Aβ sequestering led to neuronal necrosis in the early stages of AD [122]. These findings imply that STRN4 may be a potential regulator of a molecular cascade, including MAP4K and YAP involved in neuronal health and Aβ metabolism. We also found evidence through transcriptome studies that another gene at the *STRN4* locus, *PRKD2*-a protein kinase- may be the index gene. Future studies are needed to distinguish the actual functional gene at this locus.

Of the four AB40_TX_ associated loci genes, *RFX7* is the only transcription factor. Gene-based rare variant analysis of *RFX7* in the Alzheimer’s Disease Neuroimaging Initiative (ADNI) cohort showed trending significance with entorhinal cortex thickness [123]. *ITGB4*, the index gene at the Aβ40_TBS_ locus, encodes a transmembrane integrin involved in cell-to-cell adhesion, is differentially expressed in AD [124–127] with potential roles in the blood-brain barrier [128, 129], schizophrenia and bipolar disorder [130].

Though most of the novel associations were with brain Aβ40 biochemical fractions, our study also discovered two significant loci, *NPAS3* and *SCIN*, for brain APOE_TX_ levels. *NPAS3*, encoding neuronal PAS domain protein, has been implicated in neurogenesis [131], general cognitive function [132, 133], psychiatric disorders [134] like schizophrenia [135], and has protein aggregation potential [136]. *NPAS3* is also known to regulate transcriptional levels of *VGF* [137–139] which is a key regulator in protection against AD pathogenesis in 5xFAD mice models [117] and a top target identified by the AMP-AD consortium (agora.adknowledgeportal.org/). Finally, *SCIN* which encodes an actin-binding protein has variants that associate with inflammation markers [102] and rate of cognitive decline in ADs [140]. The *SCIN* locus variant associated with higher APOE_TX_ is also associated with lower Thal phase in the same cohort, suggesting that higher membrane-bound levels of APOE might have a protective role in AD. Notably, upregulated expression of *SCIN* was identified as part of a pan-neurodegenerative gene signature across AD, Lewy Body disease, and ALS-FTD [141].

In summary, investigating the functions and other genetic associations of the index genes near biochemical endophenotype GWAS loci support the conclusion that these genes and loci have functional consequences on brain health and neuropsychiatric disease. While such genetic localizations do not definitively prove the involvement of the index genes in the tested phenotypes, they nevertheless provide new testable hypotheses. Moreover, these findings underscore the potential of brain biochemical endophenotypes in the discovery of novel AD-related genes and pathways. Indeed, using the genetic association findings from our GWAS, we detected shared and distinct enrichment in GO pathways known to be important in AD risk as well as novel pathways. In general, these shared biological pathways highlight known broad biological processes in AD such as synapse organization or immune functions. In contrast, distinct pathways pinpoint processes that may relate to specific functions or biochemical states of these proteins, such as lipid metabolism for APOE and peptide cross-linking for soluble Aβ42_TBS_ and p-Tau_TBS_, respectively. This suggests that genetic influences affect not only total levels but also specific biochemical states of these AD-related proteins in the brain. These findings have implications for identifying therapeutic targets that may play a role in the transition of these proteins into pathogenic biochemical states rather than their overall levels.

In this study, we also investigated the functional mechanisms of the GWS loci by expression and splicing QTL, as well as bulk and single-cell transcriptome analyses of our and other published data. QTL analyses suggested that *STRN4*-Aβ40_TX_ rs34805055, *ITGB4*-Aβ40_TBS_ rs9890231, and *APOE/NECTIN2*-APOE_TBS_ rs283815 may modulate brain biochemical levels by impacting the expression or splicing of nearby genes*, PRKD2, ITGB4,* and *TOMM40*, respectively. Differential expression of genes at each locus revealed significant bulk or cell-specific transcriptional changes in AD for *PRKD2*, *ITGB4* and *APOE/NECTIN2/TOMM40*. Even so, colocalization analyses suggested that these signals are not a result of a single causal variant except for rs34805055-Aβ40_TX_ (*STRN4*)-*PRKD2* eQTL in the Mayo TCX dataset which had inconsistent evidence of colocalization. It should be noted, however, that many of these colocalization analyses included small datasets from healthy individuals which may not have enough power to reject the null hypothesis. Additional transcriptome studies, particularly cell type specific QTLs, are needed to further characterize the putative regulatory function of these variants.

Notably, our study identified novel GWS loci for brain biochemical levels of AD proteins that were missed in prior studies investigating CSF levels of these proteins [73] or overall neuropathology [100]. This is likely because these studies capture more global brain changes or represent combined biochemical states of these proteins. With the exception of *APOE-ε*4, none of the GWS variants identified here associated with all three biochemical fractions of a protein, suggesting that these loci likely reflect the genetic determinants for specific biochemical states of these proteins. Our findings highlight the potential for deep biochemical phenotyping and demonstrate that this approach can dissect the genetic loci and pathways involved in the specific biochemical states of AD-related proteins, which in turn has implications for understanding disease mechanisms and therapeutic development.

Our study has many strengths including extensive biochemical measures of key AD proteins Aβ, tau and APOE from three brain tissue fractions in a large sample of neuropathologically diagnosed AD patients. We validated and annotated the significant GWAS loci genes and variants by leveraging additional large-scale WGS, RNA-seq, and scRNA-seq datasets with Braak, Thal, CAA, age at death, and additional AD-related biomarker measures. We demonstrated enriched pathways that are both shared as well as those that are AD-protein and biochemical state-specific.

Nevertheless, this study has several limitations including the biochemical measures being available from a single neuropathologically-diagnosed AD cohort of 441 individuals; to our knowledge other autopsy AD cohorts with such deep brain biochemical phenotyping are lacking. Although we have leveraged other AD-related outcome associations from these and other independent samples to validate and annotate our findings, future studies in additional samples with brain biochemical measures are needed for replication as well as increased power. It should also be noted that since we used a more liberal minor allele frequency threshold of 2% and thus five of the novel GWS variants are low frequency (< 5%), we validated all novel index GWS variants with secondary confirmation methods and demonstrated high concordance with and reliability of our GWAS imputed genotypes. Nonetheless, the low frequency of these variants in combination with the relatively small GWAS sample size may influence the precision of our effect estimates. Additional studies to expand this work into larger datasets should be performed in the future to confirm these effects. Additionally, although we perform a multiple testing correction of the GWS threshold for the increased number of independent variants, we do not perform an additional correction for the number of biochemical measures tested, as these measures are not independent of each other [32]. We also note that our AD samples have other co-pathologies, as is commonly observed in neuropathologic AD. Thus, there is a possibility that these co-pathologies may have reduced the power of this study by introducing further heterogeneity. Despite this potential confounding, we were still able to achieve GWS for *APOE* and 7 novel loci. We note that of the biochemical endophenotypes analyzed, APOE and Aβ40 had GWS associations, but not tau or Aβ42. This may be because certain proteins in specific biochemical states may be under stronger genetic control vs. may have more precise measurements reflecting their true biological variability vs. a combination of these factors. Larger scale studies with increasing measurement precision may reveal genetic factors governing the other biochemical measures. Finally, this study was conducted on non-Hispanic white individuals of Northern European descent, making it necessary to expand it to individuals of non-European ancestry.

## Conclusions

Our results strongly suggest that, although the biochemical measures tested reflect proteins core to the pathology of AD, there are unique genetic loci associated with and enriched biological pathways for specific brain biochemical states of these proteins. These findings are expected to dissect the pathophysiology of the biochemical state of AD and finesse therapeutic target discovery efforts focused on these proteins. More broadly, this study presents a new approach that will be applicable to other neurodegenerative diseases to uncover novel mechanisms of proteostasis.

## Supplementary Information

**Additional file 1: Table S1.** Description of Dataset. **Table S2.** Description of Biochemical Measures. **Table S3.** Summary Statistics for SNPs with a *p*-value < 1x10E-5 in all biochemical measures. **Table S4.** Estimated proportion of biochemical variance explained by GWS index SNPs. **Table S5.** Additional association model results for the GWS loci. **Table S6.** GWAS Summary statistics for all *APOE* index variants across phenotypes. **Table S7.** Description of Independent AMP-AD and Mayo Expansion Datasets. **Table S8.** Concordance between Imputed GWAS genotypes and Taqman/Sequencing genotypes. **Table S9.** Additional Annotations of GWS loci. **Table S10.** Analysis of GWS variants in the ADNI cohort. **Table S11.** Differentially expressed genes in two or more brain regions proximal to GWS variants in the AMP-AD RNAseq datasets. **Table S12.** Cell type specific differential expression of genes proximal to GWS variants. **Table S13.** Analysis of Significant AD GWAS SNPs from Kunkel et al 2019. **Table S14.** TaqMan Assays used for genotyping key variants. **Figure S1.** Histograms of biochemical measures. **Figure S2.** Circular Manhattan Plots with no GWS SNPs. **Figure S3.** Forest Plots. **Figure S4.** Summary of gene regulatory annotations for each GWS locus. **Figure S5.** Population Substructure via Eigenstrat Analysis. **Figure S6.** Quantile-Quantile (QQ) Plots of biochemical measure GWAS. **Figure S7.** Datasets used for manuscript analytics.

## Data Availability

The data in this manuscript can be accessed via the AD Knowledge Portal, MC-CAA study. For access to content described in this manuscript see the following 10.7303/syn35564295.1. The AD Knowledge Portal is a platform for accessing data, analyses and tools generated by the Accelerating Medicines Partnership (AMP-AD) Target Discovery Program and other National Institute on Aging (NIA)-supported programs to enable open- science practices and accelerate translational learning. The data, analyses and tools are shared early in the research cycle without a publication embargo on secondary use. Data is available for general research use according to the following requirements for data access and data attribution (https://adknowledgeportal.synapse.org/DataAccess/Instructions).

## Abbreviations

A1: Tested Allele
Aβ: Amyloid-β
AD: Alzheimer’s disease
ADNI: Alzheimer’s Disease Neuroimaging Initiative
AMP-AD: Accelerating Medicines Partnership- Alzheimer’s Disease
APOE: Apolipoprotein E
APP: Amyloid Precursor Protein
β: Beta value
BIC: Bioinformatics core
BM: Brodmann Area
CAA: Cerebral amyloid angiopathy
CER: Cerebellum
CI: Confidence Interval
CNS: Central nervous system
DE: Differential expression
DEG: Differentially expressed genes
DLPFC: Dorsal lateral prefrontal cortex
eQTL: expression quantitative trait locus
FA: Formic acid soluble tissue fraction
FDR: False Discovery Rate
GAC: Genome analysis core
GBR: British in England and Scotland population
GO: Gene ontology
GWAS: Genome-wide association study
GWS: Genome-wide significant
HRC: Haplotype reference consortium
HWE: Hardy Weinberg Equilibrium
LD: Linkage disequilibrium
LOAD: Late-onset Alzheimer’s Disease
MAF: Minor Allele Frequency
MCI: Mild cognitive impairment
MRI: Magnetic Resonance Imaging
MSBB: Mount Sinai Brain Bank
N: Number
NES: Normalized Effect Size
NFT: Neurofibrillary tangles
P: *P*-value
PC: Principal Component
PET: Positron emission tomography
PHF: Paired Helical Filaments
pTau: Phosphorylated tau
QQ-plot: Quantile-quantile plot
SD: Standard Deviation
SE: Standard Error
sQTL: Splicing quantitative trait locus
ROSMAP: Religious Orders Study and Rush Memory Aging Project
scRNA-seq: Single-cell RNA sequencing
sqrt (CAA): Square root transformed CAA scores
TBS: Tris-buffered saline soluble tissue fraction
TCX: Temporal cortex
Transf: Transformation
TX: Detergent (1% Triton-X) soluble tissue fraction
WGS: Whole genome sequencing
