# Supplementary material for: Genome-wide association study of brain biochemical phenotypes reveals distinct genetic architecture of Alzheimer’s disease related proteins
Source: Mol Neurodegener. 2023 Jan 7;18:2. doi: 10.1186/s13024-022-00592-2 (PMC9825010; doi:10.1186/s13024-022-00592-2)
Supplement: Supplementary file 1 — Additional file 1: Table S1. Description of Dataset. Table S2. Description of Biochemical Measures. Table S3. Summary Statistics for SNPs with a p-value < 1x10E-5 in all biochemical measures. Table S4. Estimated proportion of biochemical variance explained by GWS index SNPs. Table S5. Additional association model results for the GWS loci. Table S6. GWAS Summary statistics for all APOE index variants across phenotypes. Table S7. Description of Independent AMP-AD and Mayo Expansion Datasets. Table S8. Concordance between Imputed GWAS genotypes and Taqman/Sequencing genotypes. Table S9. Additional Annotations of GWS loci. Table S10. Analysis of GWS variants in the ADNI cohort. Table S11. Differentially expressed genes in two or more brain regions proximal to GWS variants in the AMP-AD RNAseq datasets. Table S12. Cell type specific differential expression of genes proximal to GWS variants. Table S13. Analysis of Significant AD GWAS SNPs from Kunkel et al 2019. Table S14. TaqMan Assays used for genotyping key variants. Figure S1. Histograms of biochemical measures. Figure S2. Circular Manhattan Plots with no GWS SNPs. Figure S3. Forest Plots. Figure S4. Summary of gene regulatory annotations for each GWS locus. Figure S5. Population Substructure via Eigenstrat Analysis. Figure S6. Quantile-Quantile (QQ) Plots of biochemical measure GWAS. Figure S7. Datasets used for manuscript analytics. [file 13024_2022_592_MOESM1_ESM.zip › BioChemGWAS_SupplTextFiguresTables_FINAL.pdf]

Genome-wide association study of brain biochemical phenotypes reveals distinct genetic architecture of Alzheimer's Disease related proteins

Supplemental Text Figures and Tables

Authors:

Stephanie R. Oatman*[1] (Oatman.stephanie@mayo.edu), Joseph S. Reddy*[2] (Reddy.Joseph@mayo.edu), Zachary Quicksall[2] (Quicksall.Zachary@mayo.edu), Minerva M. Carrasquillo[1] (Carrasquillo.Minerva@mayo.edu), Xue Wang[2] (Wang.Xue@mayo.edu), Chia-Chen Liu[1] (liu.chiachen@mayo.edu), Yu Yamazaki[1] (yuyamazaki@hiroshima-u.ac.jp), Thuy T. Nguyen[1] (Nguyen.Thuy2@mayo.edu), Kimberly Malphrus[1] (kim5157@aol.com), Michael Heckman[2] (Heckman.Michael@mayo.edu), Kristi Biswas[1] (kristibiswas42@gmail.com), Kwangsik Nho[3,4,5] (knho@iupui.edu), Matthew Baker[1] (baker.matt@mayo.edu), Yuka A. Martens[1] (Martens.Yuka@mayo.edu), Na Zhao[1] (Zhao.Na@mayo.edu), Jun Pyo Kim[4] (jk166@iu.edu), Shannon L Risacher[3,4] (srisache@iupui.edu), Rosa Rademakers[1,6] (rosa.rademakers@uantwerpen.vib.be), Andrew J Saykin[3,7] (asaykin@iupui.edu), Michael DeTure[1] (DeTure.Michael@mayo.edu), Melissa E. Murray[1] (Murray.Melissa@mayo.edu), Takahisa Kanekiyo[1] (Kanekiyo.Takahisa@mayo.edu), for the Alzheimer's Disease Neuroimaging Initiative[8] (ida@loni.usc.edu), Dennis W. Dickson[1] (Dickson.Dennis@mayo.edu), Guojun Bu[1] (guojun.bu@molecularneurodegeneration.org), Mariet Allen[1] (Allen.Mariet@mayo.edu), Nilüfer Ertekin-Taner[1,9,#] (Taner.Nilufer@mayo.edu)

Author Affiliations:

1) Mayo Clinic, Department of Neuroscience, Jacksonville, FL USA
2) Mayo Clinic, Department of Quantitative Health Sciences, Jacksonville, FL USA
3) Indiana Alzheimer Disease Center, Indiana University School of Medicine, Indianapolis, IN, USA
4) Department of Radiology and Imaging Sciences, Indiana University School of Medicine, Indianapolis, IN, USA
5) School of Informatics and Computing, Indiana University School of Medicine, Indianapolis, IN, USA
6) VIB-UA Center for Molecular Neurology, VIB, University of Antwerp, Antwerp, Belgium
7) Department of Medical and Molecular Genetics, Indiana University School of Medicine, IN, USA.
8) Data used in preparation of this article were obtained from the Alzheimer's Disease Neuroimaging Initiative (ADNI) database (adni.loni.usc.edu). As such, the investigators within the ADNI contributed to the design and implementation of ADNI and/or provided data but did not participate in analysis or writing of this report. A complete listing of ADNI investigators can be found at: http://adni.loni.usc.edu/wp-content/uploads/how_to_apply/ADNI_Acknowledgement_List.pdf
9) Mayo Clinic, Department of Neurology, Jacksonville, FL USA
*Authors contributed equally
# Corresponding Author

Corresponding Author Contact Information:

Nilüfer Ertekin-Taner, MD, PhD

Mayo Clinic, Departments of Neurology and Neuroscience, 4500 San Pablo Road, Birdsall 3, Jacksonville, FL 32224. E-mail: taner.nilufer@mayo.edu, Phone: 904-953-7103, FAX: 904-953-7353.

Subset	N	N: Sex (%)		N: *APOE*-ε4 dose (%)			Mean age at death (SD)	N: Braak stage (%)			N: Thal (%)				N: Average CAA (%)			
		Male	Female	0	1	2		4	5	6	2	3	4	5	0-<1	1- <2	2-<3	3-4
**All**	441	210 (48%)	231 (52%)	145 (33%)	234 (53%)	62 (14%)	80.0 (9.1)	68 (15%)	137 (31%)	236 (54%)	3 (1%)	32 (7%)	36 (8%)	370 (84%)	283 (64%)	124 (28%)	29 (7%)	5 (1%)
***APOE*-ε4 carriers (E4+)**	296	139 (47%)	157 (53%)	0 (0%)	234 (79%)	62 (21%)	80.7 (8.6)	47 (16%)	82 (28%)	167 (56%)	1 (0%)	15 (5%)	26 (9%)	254 (86%)	181 (61%)	85 (29%)	25 (8%)	5 (2%)
***APOE*-ε4 non carriers (E4-)**	145	71 (49%)	74 (51%)	145 (100%)	0 (0%)	0 (0%)	78.7 (9.8)	21 (14%)	55 (38%)	69 (48%)	2 (1%)	17 (12%)	10 (7%)	116 (80%)	102 (70%)	39 (27%)	4 (3%)	0 (0%)

Table S1: Description of Dataset. Summary demographics of the dataset analyzed in all individuals and by *APOE*-ε4 carrier status.
N= Number, SD= Standard Deviation

Type	Extraction	Transformation	N	All		*APOE*-ε4+		*APOE*-ε4-	
				Mean	SD	Mean	SD	Mean	SD
APOE	TBS	sqrt	441	21.7	5.0	20.6	4.5	24.0	5.3
APOE	TX	sqrt	439	15.6	3.2	15.3	3.2	16.2	3.2
APOE	FA	ln	441	5.8	0.9	6.0	0.9	5.5	0.8
Aβ40	TBS	ln	439	4.3	1.6	4.4	1.6	4.0	1.5
Aβ40	TX	ln	441	5.7	0.8	5.8	0.9	5.6	0.6
Aβ40	FA	ln	441	7.3	1.7	7.5	1.8	6.8	1.6
Aβ42	TBS	ln	441	6.5	0.6	6.5	0.6	6.5	0.6
Aβ42	TX	ln	441	7.2	0.5	7.2	0.5	7.2	0.5
Aβ42	FA	ln	441	10.9	0.7	10.9	0.7	11.0	0.7
Aβ40/42	TBS	ln	439	-2.2	1.5	-2.0	1.5	-2.5	1.4
Aβ40/42	TX	ln	441	-1.4	0.8	-1.4	0.9	-1.6	0.7
Aβ40/42	FA	ln	441	-3.7	1.7	-3.4	1.7	-4.2	1.5
total tau	TBS	sqrt	441	1.9	0.8	1.9	0.8	1.8	0.9
total tau	TX	sqrt	441	36.1	11.3	35.6	10.8	36.9	12.3
total tau	FA	sqrt	438	3.3	0.9	3.3	0.9	3.3	0.9
p-tau	TBS	sqrt	441	3.2	0.8	3.2	0.8	3.2	0.8
p-tau	TX	ln	441	1.7	0.4	1.7	0.4	1.8	0.4
p-tau	FA	ln	441	7.2	1.2	7.2	1.1	7.1	1.2

Table S2: Description of Biochemical Measures. Summary statistics of all biochemical measures analyzed in all samples and stratified by *APOE*-ε4 carrier status groups. Transformations of measures done to normalize dataset for downstream analysis. N= Number, SD= Standard Deviation, sqrt= square root, ln = natural log

Table S3: SNPs with p-values < 1E-05 - See Excel sheet
Table S5: Additional association model results for the GWS loci in the absence of *APOEε*4 effects- See Excel sheet
Table S11: AMP-AD DEGs - See Excel sheet
Table S12: Mathys DEGs - See Excel sheet

Biochemical Measure	Index SNPs at GWS loci		Index SNPs at GWS loci excluding *APOE*-ε4		***APOE*-ε4 only**	
	**SNPs**	**R^2**	**SNPs**	**R^2**	**SNPs**	**R^2**
APOE TBS	rs283815, rs429358	0.104	rs283815	0.097	rs429358	0.093
APOE TX	rs116580059, rs11845003	0.123	-	-	-	-
APOE FA	-	-	-	-	rs429358	0.134
Aβ40 TBS	rs9890231	0.070	-	-	-	-
Aβ40 TX	rs116726862, rs34805055, rs148028977, rs77785770, rs429358	0.269	rs116726862, rs34805055, rs148028977, rs77785770	0.222	rs429358	0.086
Aβ40 FA	-	-	-	-	rs429358	0.124
Aβ40/42 TX	rs483082, rs429358	0.071	rs483082	0.071	rs429358	0.066
Aβ40/42 FA	-	-	-	-	rs429358	0.140

Table S4: Estimated proportion of biochemical variance explained by GWS index SNPs. The R^2 is the estimated proportion of biochemical measure variance explained by the GWS index SNPs. Proportion was estimated through linear regression models regressing index SNPs on each biochemical measure with and without *APOE*-ε4 as appropriate, and in *APOE*-ε4 only models as appropriate. Proportion of variance was estimated only for biochemical measures with GWS SNPs. SNPs= Variants included in the linear regression model, R^2= Estimated proportion of variances explained by SNPs.

		rs283815 (NECTIN2 locus)				rs429358 (*APOE4* locus)				rs483082 (*APOE;APOC1* locus)			
**Protein**	**Fraction**	**BETA**	**L95**	**U95**	**P**	**BETA**	**L95**	**U95**	**P**	**BETA**	**L95**	**U95**	**P**
APOE	TBS	-2.41	-3.10	-1.72	2.62E-11	-2.31	-3.00	-1.63	1.04E-10	-2.15	-2.82	-1.49	5.05E-10
APOE	TX	-0.38	-0.84	0.08	1.03E-01	-0.51	-0.96	-0.06	2.82E-02	-0.44	-0.88	-0.01	4.79E-02
APOE	FA	0.42	0.31	0.54	5.02E-12	0.49	0.38	0.60	4.53E-16	0.45	0.34	0.56	7.12E-15
Aβ40	TBS	0.48	0.26	0.71	2.08E-05	0.50	0.28	0.72	9.02E-06	0.42	0.21	0.64	9.98E-05
Aβ40	TX	0.30	0.18	0.42	8.20E-07	0.37	0.25	0.48	6.88E-10	0.36	0.25	0.47	7.12E-10
Aβ40	FA	0.76	0.52	1.00	1.02E-09	0.91	0.68	1.15	6.75E-14	0.85	0.62	1.07	7.87E-13
Aβ40/42	TBS	0.53	0.32	0.73	8.78E-07	0.51	0.30	0.71	1.79E-06	0.44	0.24	0.64	1.71E-05
Aβ40/42	TX	0.32	0.20	0.43	3.36E-07	0.33	0.21	0.45	7.08E-08	0.33	0.22	0.44	2.11E-08
Aβ40/42	FA	0.81	0.58	1.03	2.20E-11	0.94	0.72	1.16	1.48E-15	0.88	0.67	1.10	9.09E-15
Aβ42	TBS	-0.04	-0.13	0.05	3.79E-01	-0.01	-0.10	0.08	8.73E-01	-0.02	-0.10	0.07	6.80E-01
Aβ42	TX	-0.01	-0.09	0.06	6.96E-01	0.04	-0.03	0.11	2.67E-01	0.03	-0.04	0.10	4.48E-01
Aβ42	FA	-0.04	-0.15	0.06	4.06E-01	-0.03	-0.13	0.08	6.27E-01	-0.04	-0.14	0.06	4.64E-01
pTau	TBS	-0.05	-0.17	0.06	3.58E-01	-0.01	-0.13	0.10	8.51E-01	-0.03	-0.14	0.08	6.40E-01
pTau	TX	-0.01	-0.06	0.05	8.46E-01	-0.02	-0.07	0.04	6.04E-01	-0.01	-0.07	0.05	7.13E-01
pTau	FA	0.11	-0.05	0.28	1.74E-01	0.08	-0.08	0.24	3.18E-01	0.10	-0.06	0.25	2.28E-01
Total tau	TBS	-0.04	-0.15	0.08	5.02E-01	0.02	-0.10	0.13	7.89E-01	0.00	-0.11	0.11	9.54E-01
Total tau	TX	-1.77	-3.38	-0.16	3.13E-02	-0.64	-2.24	0.95	4.31E-01	-0.65	-2.19	0.89	4.06E-01
Total tau	FA	-0.09	-0.23	0.05	1.95E-01	-0.03	-0.17	0.10	6.32E-01	-0.06	-0.19	0.07	3.37E-01

Table S6: GWAS Summary statistics for all *APOE* index variants across phenotypes. The L95 is the lower 95% confidence interval while U95 is the upper 95% confidence interval. P is p-value.

Dataset		N	AD status		N: Sex (%)		N: *APOE-ε4* dose (%)			Mean age at death (SD)	N: Braak stage (%)							N: Thal (%)					
			AD	nonAD	Male	Female	0	1	2		0	1	2	3	4	5	6	0	1	2	3	4	5
Mayo Brain Bank Expansion		2005	1477 (74%)	528 (26%)	932 (46%)	1073 (54%)	957 (48%)	826 (41%)	218 (11%)	79.6 (8.25)	75 (4%)	62 (3%)	227 (11%)	158 (8%)	159 (8%)	410 (20%)	856 (43%)	186 (9%)	115 (6%)	49 (2%)	163 (8%)	132 (7%)	1021 (51%)
AMP-AD	Mayo	344	91 (26%)	253 (74%)	166 (48%)	178 (52%)	210 (61%)	70 (20%)	8 (2%)	81 (8.4)	18 (5%)	19 (6%)	40 (12%)	40 (12%)	6 (2%)	38 (11%)	47 (14%)	46 (13%)	27 (8%)	9 (3%)	15 (4%)	3 (1%)	57 (17%)
	MSBB	267	158 (59%)	61 (23%)	90 (34%)	177 (66%)	111 (42%)	50 (19%)	3 (1%)	83.7 (7.47)	7 (3%)	23 (9%)	35 (13%)	42 (16%)	28 (10%)	33 (12%)	99 (37%)	N/A	N/A	N/A	N/A	N/A	N/A
	ROS-MAP	1091	554 (51%)	268 (25%)	379 (35%)	712 (65%)	807 (74%)	257 (24%)	18 (2%)	86.9 (4.35)	13 (1%)	68 (6%)	98 (9%)	271 (25%)	350 (32%)	280 (26%)	11 (1%)	N/A	N/A	N/A	N/A	N/A	N/A

Table S7: Description of Independent AMP-AD and Mayo Expansion Datasets. Summary statistics of the Mayo expanded brain bank dataset and the AMP-AD WGS datasets. All cohorts had neuropathologically determined diagnoses made by experienced neuropathologists and some also had clinical data. The details of these cohorts can be found on the AD Knowledge Portal (www.synapse.org) and Methods. All datasets were used to test the association of index variants with AD diagnosis, Braak, Thal and age-at-death in a meta-analysis (Fig.3 and S3). Additionally, the Mayo expanded dataset was utilized to directly genotype the genome-wide significant index variants for confirmation of array/imputed genotypes. N= Number, SD= Standard Deviation; AMP-AD = Accelerating Partnerships in Medicine AD, MSBB= Mount Sinai Brain Bank, ROS-MAP= Religious Orders Study and Rush Memory and Aging Project.

Table S8a: All Results

Locus	*NECTIN2*		*SCIN*			*NPAS3*		*ITGB4*		*KCNN2*		*RFX7*			*SLC9A9*		*STRN4*	
SNP	rs283815		rs116580059	rs79606912 (proxy)		rs11845003		rs9890231		rs77785770		rs148028977	rs146973898 (proxy)		rs116726862		rs34805055	
Hard Call Genotype	GWAS	Taqman	GWAS	GWAS	Taqman	GWAS	Taqman	GWAS	Taqman	GWAS	Taqman	GWAS	GWAS	Taqman	GWAS	Taqman	GWAS	Sequencing
0	136	137	411	412	410	420	419	393	408	402	401	417	415	419	405	405	372	1
1	234	235	30	29	30	20	21	17	31	38	38	14	13	20	23	33	64	63
2	63	65	0	0	0	0	0	0	0	1	1	0	0	0	0	0	2	2
NA	8	4	0	0	1	1	1	31	2	0	1	10	13	2	13	3	3	-
Total N	441	441	441	441	441	441	441	441	441	441	441	441	441	441	441	441	441	0

Table S8b: Results used for Concordance Test

Hard Call Genotype	GWAS	Taqman		GWAS	Taqman	GWAS	Taqman	GWAS	Taqman	GWAS	Taqman		GWAS	Taqman	GWAS	Taqman	GWAS	Sequencing
0	136	136	-	411	410	419	418	391	387	401	401	-	413	413	402	400	0	1
1	230	229	-	29	30	20	21	17	21	38	38	-	13	13	23	25	64	63
2	63	64	-	0	0	0	0	0	0	1	1	-	0	0	0	0	2	2
NA	12	12	-	1	1	2	2	33	33	1	1	-	15	15	16	16	-	-
Total N for test:	429	429		440	440	439	439	408	408	440	440		426	426	425	425	66	66
N discordant:	1			1		1		4		0			0		2		1	
% Concordance:	428/429= 99.8%			439/440= 99.8%		438/439= 99.8%		404/408= 99.0%		440/440= 100.0%			426/426= 100.0%		423/425= 99.5%		65/66= 98.5%	

Table S8: Concordance between Imputed GWAS genotypes and Taqman/Sequencing genotypes. To compare imputed GWAS dosages with those from the Taqman assays, imputed dosages were converted to hard call genotypes where those with uncertainty >0.1 were set to missing (NA). Next, because missing genotypes cannot be utilized in a concordance analysis (i.e. both imputed hard call genotypes and TaqMan genotypes have to have a non-missing value to make a concordance comparison), only samples with a result from both the hard call imputed genotype and TaqMan genotypes were included in the concordance test. All genotypes with a missing value from either imputed GWAS hard calls (N=8) or TaqMan results (N=4) were set to NA (Supplementary Table S8b). For completeness, all results from the imputed hard call and TaqMan genotypes are reported in Supplementary Table S8a. Percent concordance was calculated for each variant by taking the number of concordant genotypes divided by the total number of samples with a non-missing result across both assays. Concordance rates for variants in which a proxy variant was used for TaqMan genotyping (SCIN and RFX7 loci) were calculated with the proxy variant imputed GWAS hard call, however, both the index variant and proxy variant results are reported below in Supplementary Table S8a. A TaqMan assay for the STRN4 locus was not available and failed design efforts, so all minor allele carriers identified from the imputed GWAS dosages for this variant were Sanger sequenced (N=66) and percent concordance was assessed similarly to the Taqman genotyped loci, however with only these minor allele carrier samples. Percent concordance for loci assessed with TaqMan assays were ≥ 99%, and that for the *STRN4* locus variant had one discordant sample with a concordance rate of 98.5%.

			Novel							Known	
		Index Variant	rs9890231	rs77785770	rs148028977	rs116726862	rs34805055	rs116580059	rs11845003	rs429358	rs283815
		Position Annotation	Intron *ITGB4*	Intron *KCNN2*	Intron *RFX7*	Intron *SLC9A9*	Intron *STRN4*	Intron *SCIN*	Intron *NPAS3*	Exon *APOE*	Intron *NECTIN2*
		Protein	Aβ40 TBS	Aβ40 TX	Aβ40 TX	Aβ40 TX	Aβ40 TX	APOE TX	APOE TX	Aβ40 TX Aβ40 FA Aβ40/42 FA APOE FA	APOE TBS
Variant level annotation	Meta-Analysis Fixed Effects- Beta (95% CI) p-value	AD Diagnosis	-0.03 (-0.27, 0.22)<br>0.84	0.01 (-0.27, 0.29)<br>0.93	0.01 (-0.39, 0.41)<br>0.96	0.4 (0.01, 0.79)<br>0.04	-0.2 (-0.51, 0.12)<br>0.22	-0.21 (-0.51, 0.09)<br>0.17	-0.23 (-0.57, 0.1)<br>0.17	1.53 (1.35, 1.7)<br>1.40E-66	0.86 (0.72, 0.99)<br>6.50E-36
		Braak stage	-0.09 (-0.27, 0.1)<br>0.37	-0.11 (-0.32, 0.1)<br>0.3	-1.8e-03 (-0.3, 0.3)<br>0.99	0.24 (-0.02, 0.5)<br>0.07	-0.14 (-0.37, 0.09)<br>0.23	-0.23 (-0.47, 9.3e-03)<br>0.06	-0.12 (-0.39, 0.14)<br>0.36	1.07 (0.96, 1.18)<br>1.80E-77	0.69 (0.59, 0.79)<br>1.20E-42
		Thal phase	0.11 (-0.2, 0.42)<br>0.48	-0.05 (-0.37, 0.27)<br>0.75	0.09 (-0.34, 0.52)<br>0.68	0.66 (0.18, 1.15)<br>7.50E-03	-0.08 (-0.8, 0.64)<br>0.83	-0.41 (-0.74, -0.08)<br>0.015	-0.24 (-0.61, 0.12)<br>0.19	1.22 (1.05, 1.38)<br>4.50E-47	0.85 (0.7, 1)<br>8.40E-29
		Age at Death	0.15 (-0.47, 0.76)<br>0.64	0.35 (-0.36, 1.06)<br>0.33	-0.61 (-1.61, 0.38)<br>0.23	0.19 (-0.65, 1.02)<br>0.66	0.02 (-0.6, 0.63)<br>0.96	0.47 (-0.35, 1.28)<br>0.26	0.64 (-0.24, 1.53)<br>0.15	-0.33 (-0.69, 0.03)<br>0.07	-0.33 (-0.64, -9.8E-03)<br>0.04
	APOE-ε2 and -ε4 Adjusted	AD Diagnosis	-5.7e-3 (-0.27, 0.26)<br>0.97	-0.05 (-0.35, 0.26)<br>0.75	0.02 (-0.42, 0.45)<br>0.93	0.5 (0.07, 0.93)<br>0.02	-0.15 (-0.5, 0.2)<br>0.4	-0.17 (-0.5, 0.16)<br>0.32	-0.24 (-0.6, 0.12)<br>0.19	-	0.08 (-0.12, 0.28)<br>0.43
		Braak stage	-0.05 (-0.25, 0.14)<br>0.58	-0.13 (-0.35, 0.09)<br>0.23	-0.03 (-0.33, 0.28)<br>0.87	0.28 (0.01, 0.56)<br>0.04	-0.08 (-0.32, 0.16)<br>0.51	-0.23 (-0.48, 0.02)<br>0.07	-0.12 (-0.39, 0.15)<br>0.38	-	0.12 (-0.03, 0.27)<br>0.12
		Thal phase	0.12 (-0.19, 0.44)<br>0.45	-0.11 (-0.45, 0.22)<br>0.51	0.11 (-0.33, 0.56)<br>0.61	0.77 (0.26, 1.27)<br>0.0028	0.06 (-0.7, 0.83)<br>0.87	-0.38 (-0.72, -0.04)<br>0.03	-0.18 (-0.56, 0.19)<br>0.34	-	0.11 (-0.12, 0.34)<br>0.37
		Age at Death	0.07 (-0.56, 0.69)<br>0.84	0.37 (-0.36, 1.09)<br>0.32	-0.57 (-1.57, 0.42)<br>0.26	0.11 (-0.73, 0.96)<br>0.79	-0.09 (-0.72, 0.55)<br>0.79	0.56 (-0.28, 1.39)<br>0.19	0.63 (-0.26, 1.53)<br>0.17	-	-0.28 (-0.76, 0.2)<br>0.26
	MC-CAA dataset associations# (N=821)	CAA (adjusted for Braak and Thal)	0.04 (5.38E-01)	0.10 (5.08E-02)	0.12 (1.07E-01)	0.13 (4.70E-02)	0.07 (6.23E-02)	0.02 (7.44E-01)	0.03 (7.09E-01)	0.18 (1.66E-16)	0.16 (6.81E-13)
	Variants in LD	Mayo TCX and CER WGS datasets (+/- 1 Mb, D'≥ 0.8 and r2 ≥0.2)	NA	NA	NA	NA	NA	rs10255616	rs113599869, rs73258992, rs58873852	rs769449, 19:45412778	NA
		1000Genomes GBR dataset (+/- 50kb, D'≥ 0.8 and r2 ≥0.8)	NA	rs78713788, rs17361210, rs115281149, rs79124698	rs555278768, rs146343566, rs138937239, rs139981434, rs138839116, rs190337649, rs146973898, rs144861372	rs115134572	rs11668878, rs62134832, rs62134819	rs148766594, rs77048498, rs79606912	Many*	rs814573, rs157592, rs12721051, rs56131196, rs4420638, rs769449, rs10414043, rs6857, rs7256200	rs283811, rs184017, rs157581, rs157582, rs59007384
		Neurological associations of LD variants from GWAS catalog search	NA	NA	NA	Survival time of sporadic ALS	NA	NA	NA	AD, blood protein levels, CAA, lipoprotein levels, Aβ and tau CSF levels	AD, CAA, Aβ42 CSF levels
Gene level annotation	GWAS catalog search for any variant within or near index gene that significantly associates with neuropsychiatric phenotypes		NA	AD- Age of onset, total amyloid (E), bipolar disorder, hippocampal sclerosis of aging, schizophrenia	Subcortical grey matter volume	Response to cholinesterase inhibitors in AD, working memory, total PHF-tau (E), ADHD, autism	NA	Macrophage inflammatory protein 1b levels	Neuritic and diffuse plaques, plasma Aβ40 levels, CSF sTREM2 levels, PHF-tau levels (E), NFT (E), schizophrenia, bipolar disorder caudate nucleus volume	Many AD related GWAS	Many AD related GWAS

Table S9: Additional Annotations of GWS loci. Variant level annotations include meta-analysis results using a fixed-effects model adjusted for sex and age at death when appropriate as well as *APOE*-ε2 and -ε4 when specified, variant associations with CAA from the MC-CAA GWAS dataset (Reddy et al. 2021), variants in LD from the AMP-AD Mayo CER and TCX WGS dataset as well as the 1000 Genomes GBR dataset, and nominally significant associations of variants in LD with neurological related traits reported in the NHGRI-EBI GWAS Catalog. Gene level annotation was performed for index genes with a GWAS catalog search for any variant with associations to neurological phenotypes. Significant associations (p-value < 0.05) are highlighted in red.

CI= Confidence Interval, CAA= Cerebral amyloid angiopathy, GBR= British in England and Scotland population, LD= Linkage disequilibrium, AMP-AD= Accelerating Medicines Partnership- AD, CER= Cerebellum, TCX= Temporal Cortex, PHF= Paired Helical Filaments, NFT= Neurofibrillary tangles. #From Reddy et al. 2021.

*Variants in LD with rs11845003 (*NPAS3*) include rs117805721, rs78458266, rs7147628, rs201012115, rs7157379, rs117768732, rs114506583, rs118166596, rs562074483, rs17101831, rs116972145, rs111312161, rs575980700, rs9671498, rs117252963, rs9671822, rs9671824, rs139661114, rs182736550, rs199850629, rs118122342, rs140879244, rs76410589, rs145358423, rs117994029, rs114136437, rs78920790, rs8021602, rs17101808, rs7143208, rs118148849, rs80342820, rs141291188, rs8011431, rs8011294, rs116994149, rs8011689, rs112209854, rs75476613, rs76847060, rs115976455, rs73258990, rs73260603, rs76540810, rs78929945, rs11851981, rs60435032, rs7161057.

SNP	Locus	A1	Original GWAS Result					Amyloid PET				CSF Amyloid				CSF p-Tau				Plasma Amyloid				Plasma p-Tau			
			Protein	Frac.	Tran	Beta	P	N	Beta	SE	P	N	Beta	SE	P	N	Beta	SE	P	N	Beta	SE	P	N	Beta	SE	P
								Adjusted for Sex + Age																			
rs116726862	*SLC9A9*	T	Aβ40	TX	ln	0.95	1.51E-08	784	-0.003	0.013	8.31E-01	1154	-0.025	0.031	4.27E-01	1151	-0.021	0.027	4.38E-01	262	0.006	0.052	9.06E-01	787	0.075	0.043	8.19E-02
rs77785770	*KCNN2*	G	Aβ40	TX	ln	0.79	3.12E-09	784	0.013	0.012	2.76E-01	1154	-0.015	0.028	5.85E-01	1151	0.018	0.024	4.56E-01	262	-0.014	0.052	7.82E-01	787	-0.016	0.041	6.99E-01
rs116580059	*SCIN*	C	APOE	TX	sqrt	3.34	1.82E-08	784	0.005	0.013	7.23E-01	1154	-0.018	0.032	5.79E-01	1151	-0.012	0.028	6.67E-01	262	0.079	0.064	2.20E-01	787	-0.034	0.043	4.25E-01
rs11845003	*NPAS3*	T	APOE	TX	sqrt	4.05	2.05E-08	784	-0.025	0.015	9.12E-02	1154	0.079	0.034	1.98E-02	1151	-0.013	0.029	6.58E-01	262	-0.062	0.053	2.41E-01	787	-0.100	0.050	4.46E-02
rs148028977	*RFX7*	T	Aβ40	TX	ln	1.24	4.47E-09	784	0.002	0.015	9.17E-01	1154	-0.035	0.035	3.15E-01	1151	0.003	0.030	9.29E-01	262	-0.010	0.078	8.94E-01	787	0.003	0.049	9.58E-01
rs9890231	*ITGB4*	G	Aβ40	TBS	ln	-1.88	2.09E-08	784	-0.016	0.009	6.98E-02	1154	0.023	0.023	3.07E-01	1151	-0.051	0.020	9.61E-03	262	-0.036	0.040	3.71E-01	787	-0.030	0.030	3.16E-01
rs283815	*NECTIN2*	G	APOE	TBS	sqrt	-2.41	2.62E-11	784	0.043	0.004	1.76E-25	1154	-0.134	0.010	8.98E-42	1151	0.083	0.009	1.12E-21	262	-0.044	0.020	2.80E-02	787	0.091	0.014	6.68E-11
rs429358	*APOE*	C	Aβ40	TX	ln	0.37	6.88E-10	784	0.057	0.004	1.14E-44	1154	-0.183	0.009	2.39E-77	1151	0.112	0.008	5.67E-37	262	-0.038	0.021	6.88E-02	787	0.124	0.014	1.96E-18
			Aβ40	FA	ln	0.91	6.75E-14																				
			Aβ40/42	FA	ln	0.94	1.48E-15																				
			APOE	FA	ln	0.49	4.53E-16																				
rs483082	*APOE;APOC1*	T	Aβ40/42	TX	ln	0.33	2.11E-08	784	0.046	0.004	1.31E-28	1154	-0.150	0.009	4.50E-51	1151	0.092	0.009	7.98E-26	262	-0.030	0.021	1.52E-01	787	0.103	0.014	2.61E-13
rs34805055	*STRN4*	T	Aβ40	TX	ln	0.61	1.15E-08	784	0.009	0.007	2.05E-01	1154	-0.011	0.018	5.28E-01	1151	0.034	0.016	2.67E-02	262	-0.005	0.034	8.90E-01	787	0.050	0.024	3.88E-02
								Adjusted for Sex + Age + *APOEε4* carrier status																			
rs116726862	*SLC9A9*	T	Aβ40	TX	ln	0.95	1.51E-08	782	-0.012	0.011	3.08E-01	1154	-0.011	0.028	6.83E-01	1151	-0.030	0.025	2.37E-01	262	0.004	0.052	9.45E-01	787	0.058	0.042	1.66E-01
rs77785770	*KCNN2*	G	Aβ40	TX	ln	0.79	3.12E-09	782	0.009	0.011	4.12E-01	1154	-0.005	0.025	8.48E-01	1151	0.012	0.023	5.90E-01	262	-0.018	0.052	7.28E-01	787	-0.019	0.039	6.18E-01
rs116580059	*SCIN*	C	APOE	TX	sqrt	3.34	1.82E-08	782	0.004	0.012	7.04E-01	1154	-0.040	0.028	1.58E-01	1151	0.003	0.026	9.20E-01	262	0.083	0.064	1.94E-01	787	-0.031	0.041	4.53E-01
rs11845003	*NPAS3*	T	APOE	TX	sqrt	4.05	2.05E-08	782	-0.016	0.013	2.28E-01	1154	0.048	0.030	1.12E-01	1151	0.007	0.027	7.86E-01	262	-0.065	0.053	2.18E-01	787	-0.084	0.048	8.01E-02
rs148028977	*RFX7*	T	Aβ40	TX	ln	1.24	4.47E-09	782	-0.014	0.013	2.97E-01	1154	0.006	0.031	8.46E-01	1151	-0.024	0.028	3.89E-01	262	-0.003	0.078	9.68E-01	787	-0.031	0.047	5.14E-01
rs9890231	*ITGB4*	G	Aβ40	TBS	ln	-1.88	2.09E-08	782	-0.011	0.008	1.64E-01	1154	0.019	0.020	3.43E-01	1151	-0.047	0.018	9.88E-03	262	-0.035	0.040	3.78E-01	787	-0.023	0.028	4.18E-01
rs283815	*NECTIN2*	G	APOE	TBS	sqrt	-2.41	2.62E-11	782	0.005	0.005	3.58E-01	1154	-0.034	0.013	9.58E-03	1151	0.013	0.012	2.64E-01	262	-0.048	0.027	7.90E-02	787	0.020	0.020	3.17E-01
rs429358	*APOE*	C	Aβ40	TX	ln	0.37	6.88E-10	782	0.031	0.009	5.95E-04	1154	-0.174	0.021	4.67E-16	1151	0.073	0.020	2.19E-04	262	-0.070	0.049	1.50E-01	787	0.105	0.033	1.56E-03
			Aβ40	FA	ln	0.91	6.75E-14																				
			Aβ40/42	FA	ln	0.94	1.48E-15																				
			APOE	FA	ln	0.49	4.53E-16																				
rs483082	*APOE;APOC1*	T	Aβ40/42	TX	ln	0.33	2.11E-08	782	0.003	0.006	5.73E-01	1154	-0.049	0.015	1.48E-03	1151	0.017	0.014	2.18E-01	262	-0.021	0.033	5.27E-01	787	0.034	0.022	1.21E-01
rs34805055	*STRN4*	T	Aβ40	TX	ln	0.61	1.15E-08	782	0.007	0.006	2.74E-01	1154	-0.005	0.016	7.75E-01	1151	0.030	0.015	3.99E-02	262	-0.001	0.034	9.73E-01	787	0.049	0.023	3.46E-02

Table S10: Analysis of GWS variants in the ADNI cohort. Association results of GWS variants with five endophenotypes including amyloid PET, CSF levels of amyloid and p-Tau, and levels of plasma amyloid and tau. Models were adjusted for sex and age, as well as *APOEε4* carrier status when specified. Nominally significant associations with P < 0.05 are in red text and those with 0.05 < P < 0.1 are in dark red text. A1= Tested allele, Frac.= Tissue Fraction, Tran= Transformation, SE= Standard Error, P= P-value.

Locus	Kunkle β	Protein Fraction SNP	A1	Aβ40 TBS β	Aβ40 TBS P	Aβ40 TX β	Aβ40 TX P	Aβ40 FA β	Aβ40 FA P	Aβ42 TBS β	Aβ42 TBS P	Aβ42 TX β	Aβ42 TX P	Aβ42 FA β	Aβ42 FA P	Aβ40/42 TBS β	Aβ40/42 TBS P	Aβ40/42 TX β	Aβ40/42 TX P	Aβ40/42 FA β	Aβ40/42 FA P
SORL1	-0.21	rs11218343	C	0.2	5.35E-01	0	9.85E-01	-0.18	6.17E-01	0	9.78E-01	-0.04	6.99E-01	-0.17	2.51E-01	0.21	5.06E-01	0.04	8.06E-01	-0.01	9.81E-01
CASS4	-0.13	rs6024870	A	0.06	7.70E-01	-0.06	5.33E-01	0.05	7.99E-01	0.03	7.05E-01	0.01	8.86E-01	0.06	4.75E-01	0.03	8.61E-01	-0.07	4.84E-01	-0.01	9.65E-01
CLU	-0.13	rs9331896	C	0.15	1.74E-01	0.06	3.18E-01	0.01	9.43E-01	-0.02	6.49E-01	0.01	7.53E-01	-0.07	1.46E-01	0.17	1.09E-01	0.05	4.23E-01	0.08	4.86E-01
PICALM; EED	-0.12	rs3851179	T	0.03	7.78E-01	-0.05	3.84E-01	0.05	6.83E-01	0.02	6.70E-01	0.03	4.03E-01	-0.02	7.56E-01	0.01	9.28E-01	-0.08	1.76E-01	0.07	5.79E-01
MS4A2;MS4A6A	-0.12	rs7933202	C	0.11	3.67E-01	0.07	2.37E-01	0.12	3.56E-01	-0.02	6.70E-01	-0.01	8.08E-01	0	9.70E-01	0.13	2.43E-01	0.08	1.90E-01	0.12	3.50E-01
EPHA1	-0.10	rs10808026	A	-0.1	4.61E-01	0.01	8.72E-01	-0.06	6.86E-01	-0.08	1.26E-01	-0.09	3.43E-02	-0.14	2.98E-02	-0.02	8.72E-01	0.11	1.61E-01	0.08	6.08E-01
INPP5D	-0.10	rs10933431	G	0.08	5.59E-01	-0.02	7.38E-01	0	9.86E-01	0.01	8.09E-01	-0.04	3.57E-01	-0.04	4.80E-01	0.06	6.19E-01	0.02	8.32E-01	0.04	7.76E-01
SPI1	-0.09	rs3740688	G	-0.01	9.45E-01	0.02	7.32E-01	-0.02	8.35E-01	-0.04	3.97E-01	-0.04	2.70E-01	-0.05	2.84E-01	0.03	7.86E-01	0.06	3.23E-01	0.03	8.07E-01
SLC24A4	-0.09	rs12881735	C	0.16	2.04E-01	0.1	1.34E-01	0.09	5.26E-01	0.01	9.07E-01	0.01	7.73E-01	-0.04	4.77E-01	0.16	1.93E-01	0.09	1.91E-01	0.13	3.38E-01
CYYR1; ADAMTS1	-0.08	rs2830500	A	0.07	5.30E-01	0.03	6.18E-01	0.03	8.27E-01	0.03	4.95E-01	0.02	6.01E-01	-0.01	8.97E-01	0.04	6.90E-01	0.01	8.54E-01	0.03	7.79E-01
IQCK	-0.08	rs7185636	C	0.3	2.75E-02	0.07	3.46E-01	0.09	5.59E-01	0.01	8.25E-01	0.02	5.80E-01	0.01	9.21E-01	0.29	2.44E-02	0.04	5.46E-01	0.08	5.75E-01
NYAP1	-0.07	rs12539172	T	0.11	3.66E-01	0.06	3.35E-01	0.35	7.02E-03	-0.01	8.64E-01	0	9.81E-01	0.01	8.10E-01	0.12	3.02E-01	0.06	3.49E-01	0.34	7.42E-03
ADAM10;LOC101928725	-0.06	rs593742	G	-0.09	4.39E-01	-0.03	6.07E-01	-0.14	2.78E-01	-0.08	9.50E-02	-0.06	1.43E-01	-0.01	7.97E-01	-0.01	9.03E-01	0.02	7.23E-01	-0.13	3.14E-01
MEF2C-AS1	-0.06	rs190982	G	-0.01	9.47E-01	0.03	6.36E-01	-0.07	5.26E-01	0.03	5.45E-01	0.01	7.93E-01	-0.03	5.67E-01	-0.03	7.49E-01	0.02	7.55E-01	-0.05	6.83E-01
GPR141; NME8	-0.05	rs4723711	T	-0.04	7.41E-01	-0.01	8.89E-01	0.05	6.91E-01	-0.02	7.38E-01	-0.02	5.88E-01	-0.02	6.96E-01	-0.02	8.26E-01	0.01	8.57E-01	0.07	5.63E-01
USP6NL; ECHDC3	0.08	rs7920721	G	-0.15	1.67E-01	-0.05	3.79E-01	-0.08	5.08E-01	-0.01	7.51E-01	-0.01	7.01E-01	0.06	1.91E-01	-0.13	1.86E-01	-0.04	5.21E-01	-0.14	2.13E-01
TNFRSF21; CD2AP	0.08	rs9473117	C	0.07	5.72E-01	0	9.98E-01	0.12	3.71E-01	0.02	6.85E-01	-0.04	2.85E-01	-0.05	3.67E-01	0.05	6.39E-01	0.04	5.28E-01	0.17	1.90E-01
PTK2B	0.09	rs73223431	T	0.1	3.23E-01	0	9.86E-01	0.05	6.79E-01	0.02	5.66E-01	0.05	1.25E-01	0.08	8.13E-02	0.08	4.04E-01	-0.05	3.58E-01	-0.04	7.50E-01
FERMT2	0.12	rs17125924	G	-0.13	4.89E-01	0.14	1.85E-01	0.22	3.03E-01	0.05	4.93E-01	0.04	5.04E-01	0.07	4.33E-01	-0.18	3.02E-01	0.09	3.60E-01	0.15	4.68E-01
ABCA7	0.12	rs3752246	G	-0.06	6.66E-01	0.01	9.04E-01	-0.01	9.63E-01	0	9.45E-01	-0.03	4.92E-01	0.02	7.48E-01	-0.07	6.33E-01	0.04	6.00E-01	-0.03	8.52E-01
CR1	0.15	rs4844610	A	0.1	4.60E-01	0.02	7.77E-01	0.14	3.70E-01	0.02	7.72E-01	-0.01	8.25E-01	0.02	7.50E-01	0.09	5.00E-01	0.03	6.82E-01	0.12	4.31E-01
WWOX; MAF	0.15	rs62039712	A	-0.03	8.75E-01	0.09	4.18E-01	0.14	5.33E-01	-0.04	6.07E-01	-0.1	1.20E-01	-0.16	8.79E-02	0.01	9.72E-01	0.19	8.64E-02	0.3	1.69E-01
BIN1; CYP27C1	0.17	rs6733839	T	0.05	6.37E-01	0.03	6.04E-01	0.07	5.19E-01	0.02	6.41E-01	0.04	2.00E-01	0.02	6.20E-01	0.03	7.70E-01	-0.01	8.08E-01	0.05	6.51E-01
CYB561; ACE	0.25	rs138190086	A	-0.02	9.54E-01	-0.06	7.75E-01	0.24	5.45E-01	0.03	8.48E-01	0.03	7.68E-01	-0.05	7.58E-01	-0.05	8.81E-01	-0.09	6.49E-01	0.29	4.50E-01
OARD1	0.30	rs114812713	C	-0.41	1.37E-01	0.03	8.62E-01	-0.16	6.02E-01	-0.18	9.34E-02	-0.06	4.73E-01	-0.13	3.03E-01	-0.23	3.78E-01	0.09	5.53E-01	-0.03	9.23E-01
APOE	1.20	rs429358	C	0.5	9.02E-06	0.37	6.88E-10	0.91	6.75E-14	-0.01	8.73E-01	0.04	2.67E-01	-0.03	6.27E-01	0.51	1.79E-06	0.33	7.08E-08	0.94	1.48E-15

Locus	Kunkle β	Protein Fraction SNP	A1	APOE TBS β	APOE TBS P	APOE TX β	APOE TX P	APOE FA β	APOE FA P	Total tau TBS β	Total tau TBS P	Total tau TX β	Total tau TX P	Total tau FA β	Total tau FA P	p-Tau TBS β	p-Tau TBS P	p-Tau TX β	p-Tau TX P	p-Tau FA β	p-Tau FA P
SORL1	-0.21	rs11218343	C	1.3	2.22E-01	1.53	2.29E-02	-0.1	5.90E-01	0.08	6.48E-01	1.73	4.64E-01	0.11	5.80E-01	0.28	9.18E-02	-0.02	8.24E-01	-0.2	4.02E-01
CASS4	-0.13	rs6024870	A	0.21	7.31E-01	0.01	9.76E-01	0.04	6.74E-01	0.13	1.88E-01	-0.34	8.01E-01	0.08	4.70E-01	-0.14	1.35E-01	-0.07	1.35E-01	-0.03	8.19E-01
CLU	-0.13	rs9331896	C	0.75	3.36E-02	0.54	1.63E-02	-0.03	5.88E-01	0.12	3.17E-02	1.25	1.14E-01	-0.03	6.27E-01	0.03	5.83E-01	-0.07	1.50E-02	-0.16	5.11E-02
PICALM; EED	-0.12	rs3851179	T	-0.27	4.47E-01	-0.22	3.28E-01	-0.01	8.75E-01	-0.01	9.26E-01	-0.44	5.75E-01	0.1	1.45E-01	-0.1	9.09E-02	-0.03	2.76E-01	0.05	5.22E-01
MS4A2;MS4A6A	-0.12	rs7933202	C	0.07	8.60E-01	-0.11	6.57E-01	0.01	8.53E-01	-0.01	8.25E-01	-0.76	3.67E-01	-0.06	4.26E-01	0.08	2.00E-01	0	9.47E-01	-0.02	7.85E-01
EPHA1	-0.10	rs10808026	A	-0.35	4.31E-01	0.05	8.63E-01	-0.03	6.91E-01	-0.05	4.81E-01	-1.05	2.90E-01	-0.23	5.15E-03	-0.05	4.40E-01	0	9.39E-01	-0.07	5.07E-01
INPP5D	-0.10	rs10933431	G	0	9.97E-01	0.01	9.77E-01	0.06	4.15E-01	-0.03	7.08E-01	0.26	7.90E-01	0.06	4.29E-01	-0.11	1.03E-01	0.01	7.90E-01	0.07	4.73E-01
SPI1	-0.09	rs3740688	G	0.19	5.76E-01	0.01	9.69E-01	-0.07	2.04E-01	0	9.96E-01	-0.51	5.09E-01	-0.05	4.40E-01	-0.05	3.62E-01	0	8.72E-01	0.01	8.62E-01
SLC24A4	-0.09	rs12881735	C	-0.26	5.22E-01	0.16	5.35E-01	-0.01	8.96E-01	0.02	8.09E-01	0.16	8.65E-01	-0.05	5.18E-01	0	9.84E-01	-0.02	5.79E-01	-0.06	5.46E-01
CYYR1; ADAMTS1	-0.08	rs2830500	A	-0.31	3.89E-01	-0.41	7.57E-02	-0.04	5.26E-01	-0.07	2.10E-01	-1.03	2.01E-01	-0.09	1.63E-01	-0.01	8.70E-01	0	9.09E-01	-0.08	3.24E-01
IQCK	-0.08	rs7185636	C	0.56	2.01E-01	0.32	2.48E-01	0.09	2.32E-01	0.05	4.96E-01	0.57	5.56E-01	0.18	2.73E-02	-0.07	2.96E-01	-0.03	3.48E-01	0.1	2.97E-01
NYAP1	-0.07	rs12539172	T	-0.09	8.16E-01	0.24	3.13E-01	0.08	2.24E-01	-0.01	8.76E-01	-0.14	8.71E-01	-0.06	3.73E-01	-0.12	5.38E-02	-0.01	7.65E-01	-0.03	7.58E-01
ADAM10;LOC101928725	-0.06	rs593742	G	0.08	8.29E-01	0.23	3.39E-01	0	9.56E-01	0.12	4.02E-02	1.41	9.67E-02	-0.13	5.96E-02	-0.03	5.97E-01	-0.04	2.40E-01	-0.18	3.50E-02
MEF2C-AS1	-0.06	rs190982	G	0.24	4.75E-01	0.17	4.31E-01	-0.05	3.97E-01	0.08	1.40E-01	0.51	4.95E-01	0.04	4.99E-01	-0.06	2.58E-01	-0.03	2.35E-01	0.04	6.14E-01
GPR141; NME8	-0.05	rs4723711	T	-0.28	4.56E-01	-0.28	2.35E-01	0.07	2.64E-01	0.01	8.07E-01	-0.12	8.87E-01	0.04	5.83E-01	-0.08	1.60E-01	-0.03	3.76E-01	0.01	9.13E-01
USP6NL; ECHDC3	0.08	rs7920721	G	0.32	3.40E-01	0	9.83E-01	0	9.80E-01	0.06	2.87E-01	-0.3	6.87E-01	0	9.50E-01	-0.06	3.06E-01	-0.01	7.68E-01	-0.03	7.30E-01
TNFRSF21; CD2AP	0.08	rs9473117	C	0.15	6.99E-01	-0.46	5.91E-02	0.01	8.87E-01	-0.05	3.97E-01	0.24	7.79E-01	0.03	6.35E-01	-0.04	4.93E-01	0.01	8.43E-01	0.15	9.09E-02
PTK2B	0.09	rs73223431	T	-0.21	5.27E-01	-0.1	6.27E-01	-0.04	4.82E-01	-0.05	3.37E-01	-0.13	8.58E-01	0.01	8.84E-01	-0.03	5.69E-01	0.06	2.43E-02	0.19	9.75E-03
FERMT2	0.12	rs17125924	G	0.45	4.57E-01	-0.02	9.60E-01	0.03	7.45E-01	0	9.66E-01	-1.51	2.65E-01	-0.09	4.43E-01	0	9.84E-01	0.01	7.81E-01	0.08	5.61E-01
ABCA7	0.12	rs3752246	G	0.32	4.93E-01	0.2	5.08E-01	0.04	6.06E-01	0.07	3.70E-01	-0.5	6.35E-01	-0.1	2.47E-01	0.06	4.43E-01	-0.02	6.85E-01	-0.04	7.01E-01
CR1	0.15	rs4844610	A	-0.63	1.55E-01	-0.73	9.45E-03	-0.01	8.73E-01	-0.09	1.99E-01	-1.15	2.44E-01	0.03	7.60E-01	-0.06	4.15E-01	0.01	7.44E-01	0.05	6.09E-01
WWOX; MAF	0.15	rs62039712	A	0.59	3.61E-01	0.08	8.50E-01	0.08	4.42E-01	0.16	1.08E-01	2.03	1.59E-01	0.01	9.24E-01	-0.14	1.75E-01	-0.09	9.08E-02	-0.09	5.27E-01
BIN1; CYP27C1	0.17	rs6733839	T	-0.53	1.11E-01	-0.28	1.85E-01	-0.06	2.97E-01	-0.05	2.99E-01	-0.52	4.88E-01	-0.01	8.95E-01	-0.01	8.50E-01	0.03	2.50E-01	0.08	2.73E-01
CYB561; ACE	0.25	rs138190086	A	0.59	6.10E-01	-0.36	6.24E-01	0.17	3.96E-01	-0.09	6.44E-01	-0.88	7.33E-01	0.13	5.57E-01	0.04	8.40E-01	0.03	7.54E-01	0.13	6.14E-01
OARD1	0.30	rs114812713	C	-0.03	9.76E-01	0.07	9.06E-01	0.16	2.99E-01	-0.12	3.81E-01	-2.56	1.91E-01	-0.12	4.83E-01	0.02	8.60E-01	0.14	4.78E-02	0.19	3.29E-01
APOE	1.20	rs429358	C	-2.31	1.04E-10	-0.51	2.82E-02	0.49	4.53E-16	0.02	7.89E-01	-0.64	4.31E-01	-0.03	6.32E-01	-0.01	8.51E-01	-0.02	6.04E-01	0.08	3.18E-01

Table S13: Analysis of Significant AD GWAS SNPs from Kunkel et al 2019. Results of significant SNPs identified by Kunkel et al. 2019 in their meta-analyses for each biochemical fraction including their beta estimate which was aligned with our GWAS tested allele (A1). Of the 28 SNPs identified in Kunkel et al. 2019, 26 were present in our dataset. The two SNPs not present in our dataset include rs75932628 (*TREM2*) and rs9271058 (*HLA-DRB1*). Ten SNPs were significant (p-value < 0.05) in at least one biochemical fraction while eight associations with the *APOEε4* tagging variant survived Bonferroni correction for 26 tests (p-value < 1.92E-03). Associations with a p-values < 0.05 are outlined in red color for a negative beta or blue color for a positive beta. Associations that survive Bonferroni correction (p-values < 1.92E-03) have the above and positive betas filled blue and negative betas filled red. A1= Tested allele, β= Beta value, P= P-value.

	rsID	Proxy rsID	Taqman assay ID
**1**	rs11845003	-	C__32007925_10
**2**	rs116726862	-	C_150768532_10
**3**	rs77785770	-	C_105058705_10
**4**	rs9890231	-	ANFVZWM
**5**	rs283815	-	C_188843436_10
**6**	rs116580059	rs79606912	C_100730255_10
**7**	rs148028977	rs146973898	C_163754645_10
**8**	rs429358	-	C___3084793_20

Table S14: TaqMan Assays used for genotyping key variants. IDs for TaqMan assays used to genotype key variants or their proxies (r^2 = 1, D' = 1 in 1000 Genomes EUR). Variants 1- 7 were genotyped for this study, variant 8 was genotyped previously using this assay and queried from an in-house database.

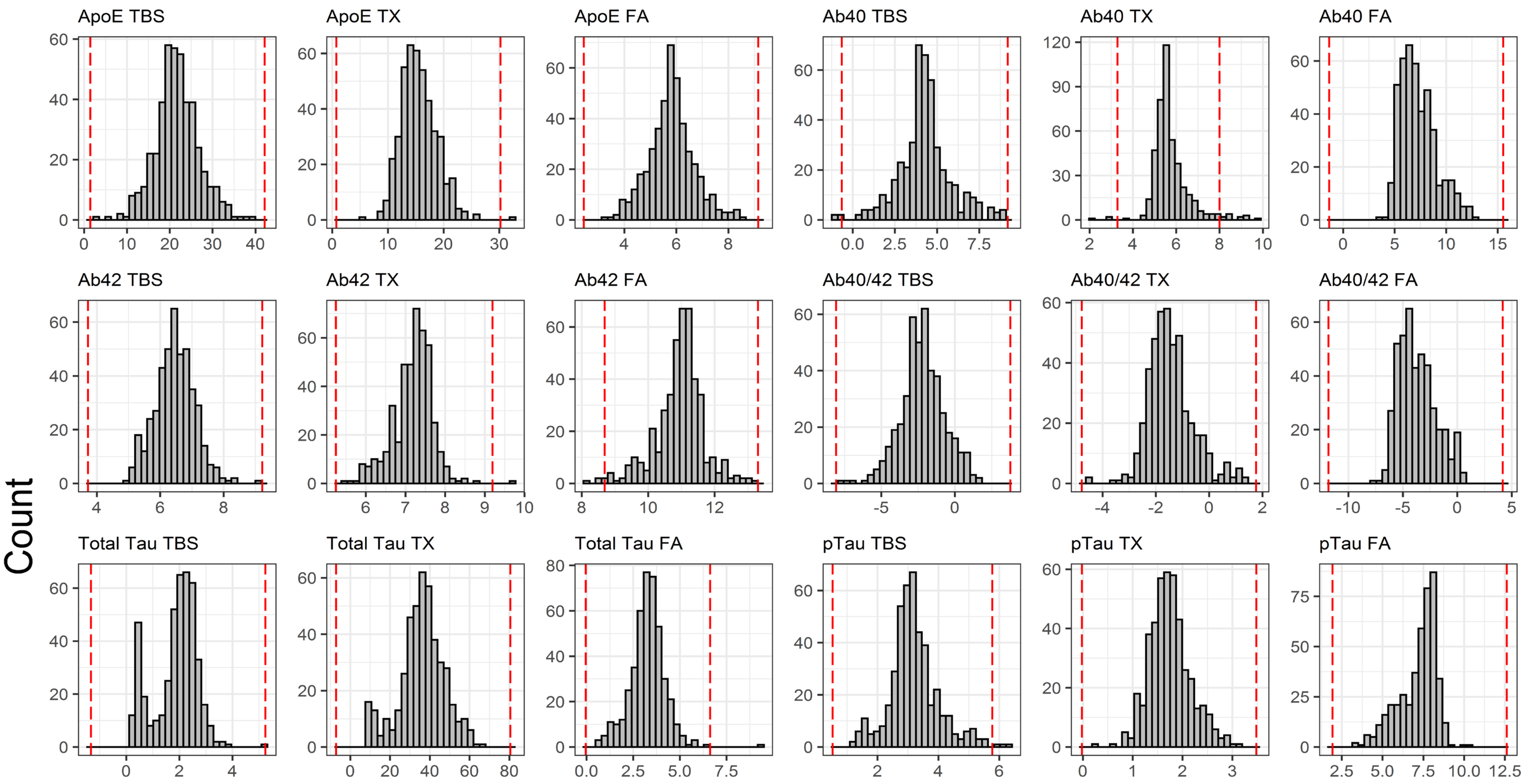

Figure S1: Histograms of biochemical measures. Histogram plots of all transformed biochemical measures. Dashed red lines define 3 times the interquartile range.

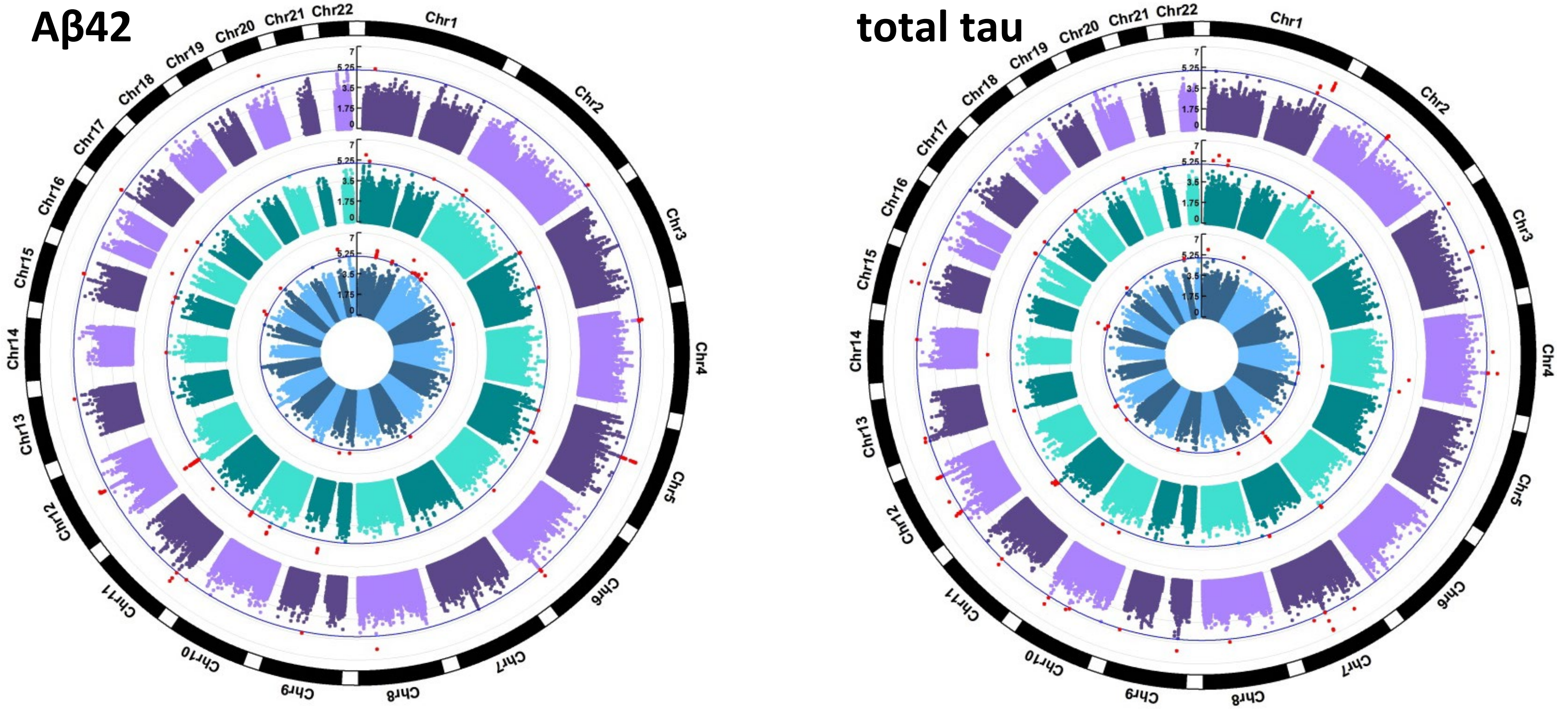

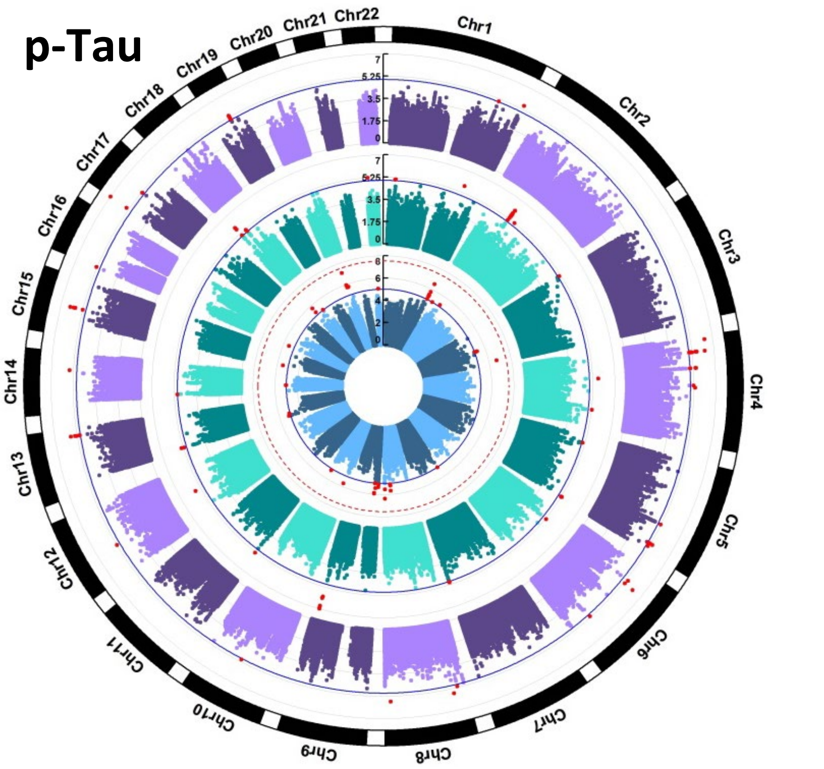

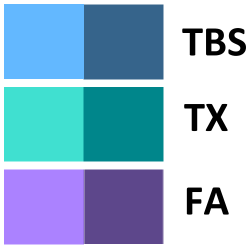

Figure S2: Circular Manhattan Plots with no GWS SNPs. Circular Manhattan plots for proteins and fractions with no genome-wide significance SNPs (p-value < 2.98E-08). Solid blue line marks p-value = 1E-05, red dotted line marks p-value = 2.98E-08. SNPs with a p-value < 1E-05 are colored red. Radial axes measure -log10(p-value). Inner most blue circle is the soluble TBS fraction, middle green circle is the membrane TX fraction and outer most purple circle is the insoluble FA fraction.

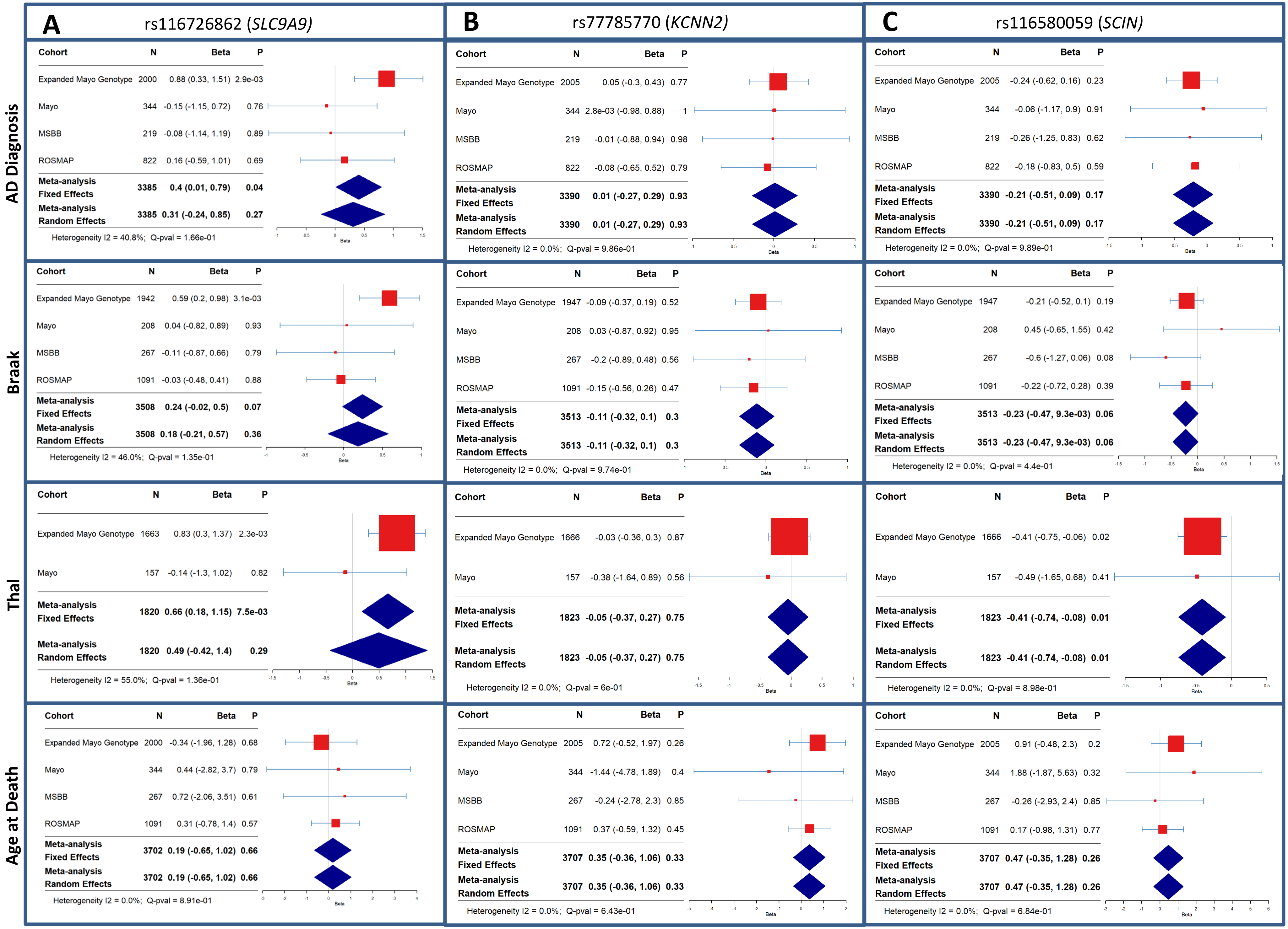

Figure S3: Forest Plots. Forest plots of GWS index variant associations and meta-analysis results (A-I) in independent AMP-AD and expanded Mayo genotype datasets with available AD related measures. Regression analyses include logistic for AD diagnosis, ordinal for Braak stage and Thal phase, and linear for Age at Death association tests (red boxes). Inverse variance weighted meta-analyses performed with fixed and random effects (blue diamonds).

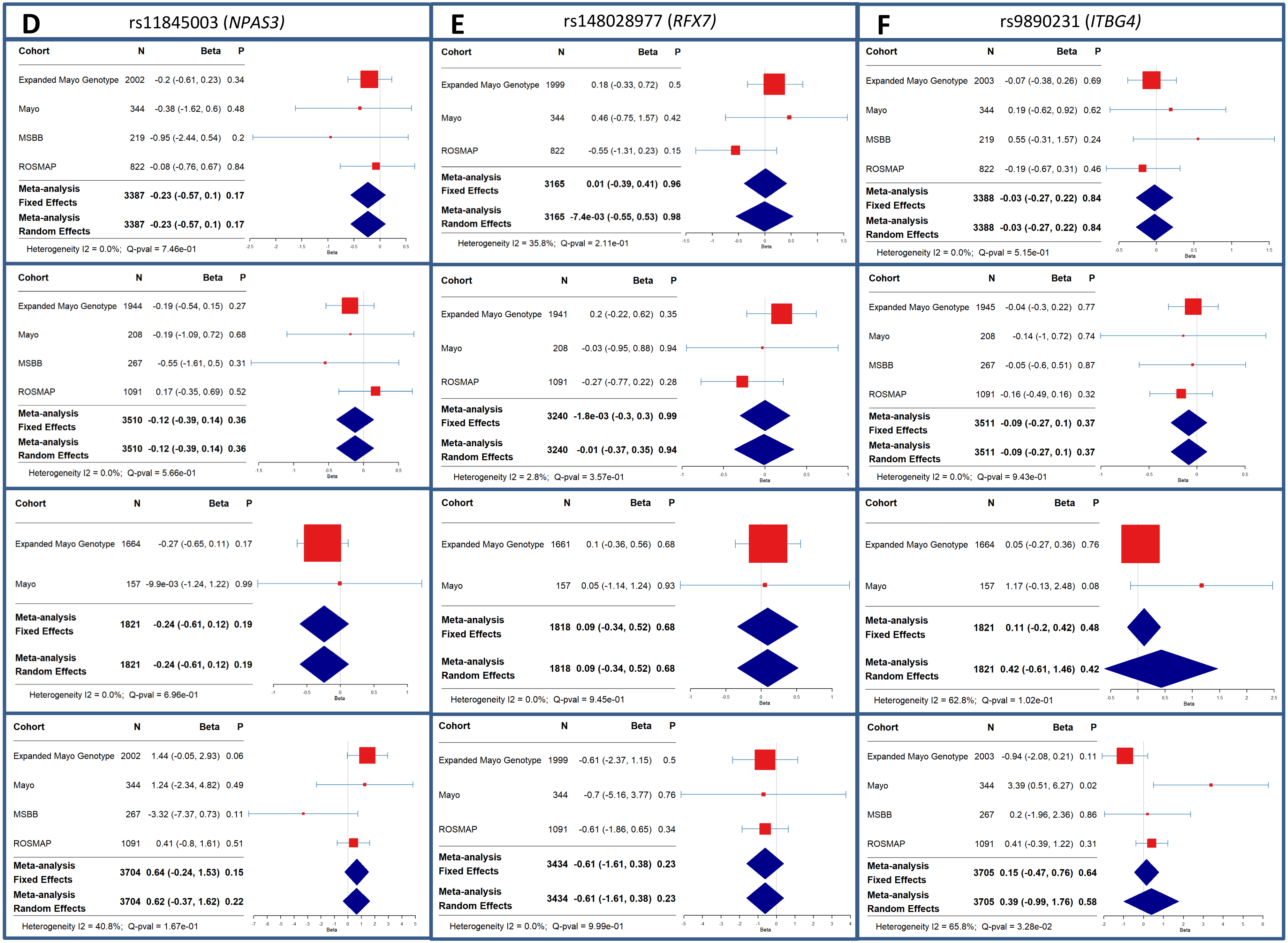
D
rs11845003 (NPAS3)
E
rs148028977 (RFX7)
F
rs9890231 (ITBG4)
AD Diagnosis
Braak
Thal
Age at Death
Cohort N Beta P
Expanded Mayo Genotype 2002 -0.2 (-0.61, 0.23) 0.34
Mayo 344 -0.38 (-1.62, 0.6) 0.48
MSBB 219 -0.95 (-2.44, 0.54) 0.2
ROSMAP 822 -0.08 (-0.76, 0.67) 0.84
Meta-analysis Fixed Effects 3387 -0.23 (-0.57, 0.1) 0.17
Meta-analysis Random Effects 3387 -0.23 (-0.57, 0.1) 0.17
Heterogeneity I2 = 0.0%; Q-pval = 7.46e-01
Beta
Cohort N Beta P
Expanded Mayo Genotype 1999 0.18 (-0.33, 0.72) 0.5
Mayo 344 0.46 (-0.75, 1.57) 0.42
ROSMAP 822 -0.55 (-1.31, 0.23) 0.15
Meta-analysis Fixed Effects 3165 0.01 (-0.39, 0.41) 0.96
Meta-analysis Random Effects 3165 -7.4e-03 (-0.55, 0.53) 0.98
Heterogeneity I2 = 35.8%; Q-pval = 2.11e-01
Beta
Cohort N Beta P
Expanded Mayo Genotype 2003 -0.07 (-0.38, 0.26) 0.69
Mayo 344 0.19 (-0.62, 0.92) 0.62
MSBB 219 0.55 (-0.31, 1.57) 0.24
ROSMAP 822 -0.19 (-0.67, 0.31) 0.46
Meta-analysis Fixed Effects 3388 -0.03 (-0.27, 0.22) 0.84
Meta-analysis Random Effects 3388 -0.03 (-0.27, 0.22) 0.84
Heterogeneity I2 = 0.0%; Q-pval = 5.15e-01
Beta
Cohort N Beta P
Expanded Mayo Genotype 1944 -0.19 (-0.54, 0.15) 0.27
Mayo 208 -0.19 (-1.09, 0.72) 0.68
MSBB 267 -0.55 (-1.61, 0.5) 0.31
ROSMAP 1091 0.17 (-0.35, 0.69) 0.52
Meta-analysis Fixed Effects 3510 -0.12 (-0.39, 0.14) 0.36
Meta-analysis Random Effects 3510 -0.12 (-0.39, 0.14) 0.36
Heterogeneity I2 = 0.0%; Q-pval = 5.66e-01
Beta
Cohort N Beta P
Expanded Mayo Genotype 1941 0.2 (-0.22, 0.62) 0.35
Mayo 208 -0.03 (-0.95, 0.88) 0.94
ROSMAP 1091 -0.27 (-0.77, 0.22) 0.28
Meta-analysis Fixed Effects 3240 -1.8e-03 (-0.3, 0.3) 0.99
Meta-analysis Random Effects 3240 -0.01 (-0.37, 0.35) 0.94
Heterogeneity I2 = 2.8%; Q-pval = 3.57e-01
Beta
Cohort N Beta P
Expanded Mayo Genotype 1945 -0.04 (-0.3, 0.22) 0.77
Mayo 208 -0.14 (-1, 0.72) 0.74
MSBB 267 -0.05 (-0.6, 0.51) 0.87
ROSMAP 1091 -0.16 (-0.49, 0.16) 0.32
Meta-analysis Fixed Effects 3511 -0.09 (-0.27, 0.1) 0.37
Meta-analysis Random Effects 3511 -0.09 (-0.27, 0.1) 0.37
Heterogeneity I2 = 0.0%; Q-pval = 9.43e-01
Beta
Cohort N Beta P
Expanded Mayo Genotype 1664 -0.27 (-0.65, 0.11) 0.17
Mayo 157 -9.9e-03 (-1.24, 1.22) 0.99
Meta-analysis Fixed Effects 1821 -0.24 (-0.61, 0.12) 0.19
Meta-analysis Random Effects 1821 -0.24 (-0.61, 0.12) 0.19
Heterogeneity I2 = 0.0%; Q-pval = 6.96e-01
Beta
Cohort N Beta P
Expanded Mayo Genotype 1661 0.1 (-0.36, 0.56) 0.68
Mayo 157 0.05 (-1.14, 1.24) 0.93
Meta-analysis Fixed Effects 1818 0.09 (-0.34, 0.52) 0.68
Meta-analysis Random Effects 1818 0.09 (-0.34, 0.52) 0.68
Heterogeneity I2 = 0.0%; Q-pval = 9.45e-01
Beta
Cohort N Beta P
Expanded Mayo Genotype 1664 0.05 (-0.27, 0.36) 0.76
Mayo 157 1.17 (-0.13, 2.48) 0.08
Meta-analysis Fixed Effects 1821 0.11 (-0.2, 0.42) 0.48
Meta-analysis Random Effects 1821 0.42 (-0.61, 1.46) 0.42
Heterogeneity I2 = 62.8%; Q-pval = 1.02e-01
Beta
Cohort N Beta P
Expanded Mayo Genotype 2002 1.44 (-0.05, 2.93) 0.06
Mayo 344 1.24 (-2.34, 4.82) 0.49
MSBB 267 -3.32 (-7.37, 0.73) 0.11
ROSMAP 1091 0.41 (-0.8, 1.61) 0.51
Meta-analysis Fixed Effects 3704 0.64 (-0.24, 1.53) 0.15
Meta-analysis Random Effects 3704 0.62 (-0.37, 1.62) 0.22
Heterogeneity I2 = 40.8%; Q-pval = 1.67e-01
Beta
Cohort N Beta P
Expanded Mayo Genotype 1999 -0.61 (-2.37, 1.15) 0.5
Mayo 344 -0.7 (-5.16, 3.77) 0.76
ROSMAP 1091 -0.61 (-1.86, 0.65) 0.34
Meta-analysis Fixed Effects 3434 -0.61 (-1.61, 0.38) 0.23
Meta-analysis Random Effects 3434 -0.61 (-1.61, 0.38) 0.23
Heterogeneity I2 = 0.0%; Q-pval = 9.99e-01
Beta
Cohort N Beta P
Expanded Mayo Genotype 2003 -0.94 (-2.08, 0.21) 0.11
Mayo 344 3.39 (0.51, 6.27) 0.02
MSBB 267 0.2 (-1.96, 2.36) 0.86
ROSMAP 1091 0.41 (-0.39, 1.22) 0.31
Meta-analysis Fixed Effects 3705 0.15 (-0.47, 0.76) 0.64
Meta-analysis Random Effects 3705 0.39 (-0.99, 1.76) 0.58
Heterogeneity I2 = 65.8%; Q-pval = 3.28e-02
Beta

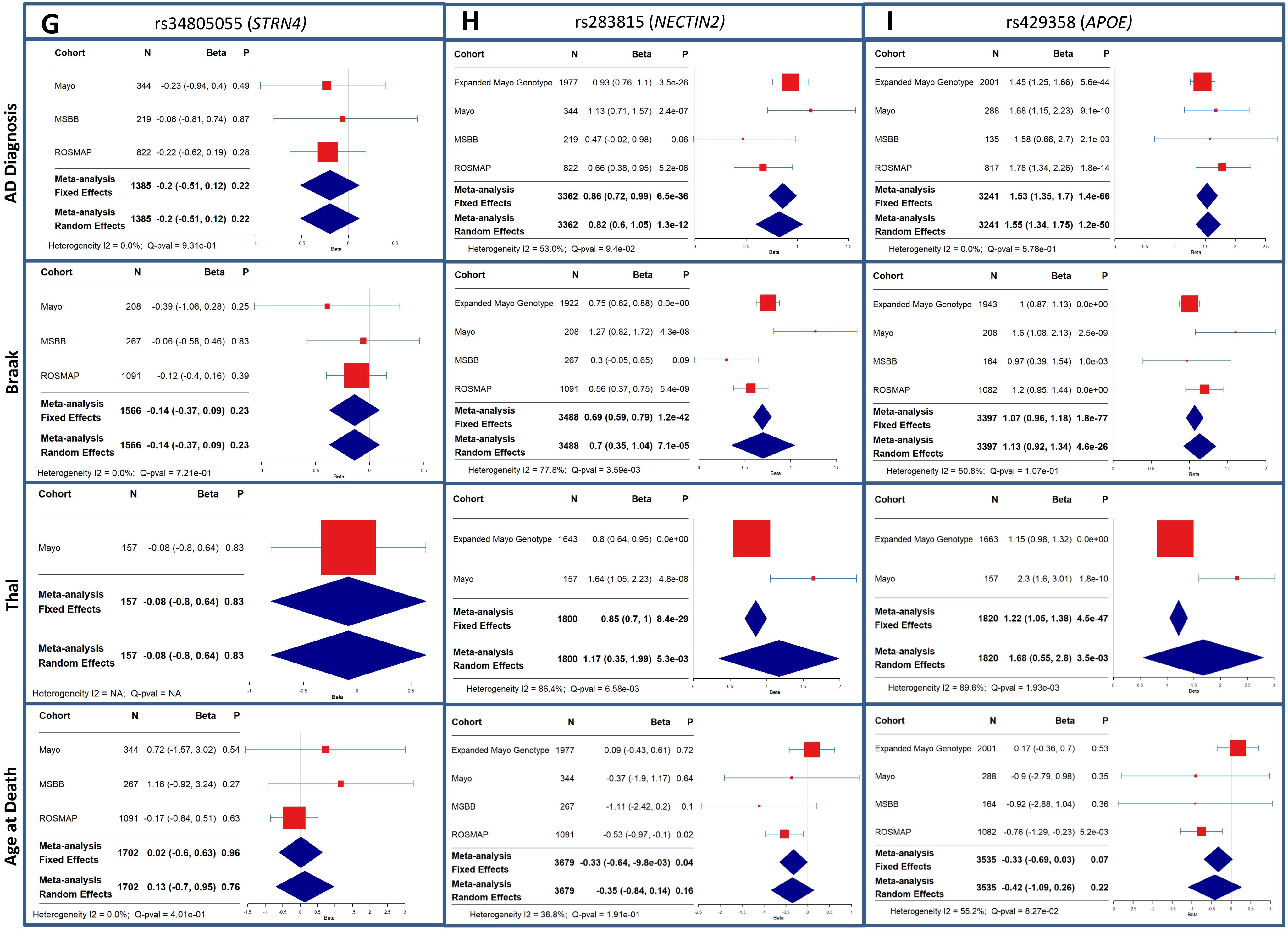
G
rs34805055 (STRN4)
H
rs283815 (NECTIN2)
I
rs429358 (APOE)
AD Diagnosis
Cohort N Beta P
Mayo 344 -0.23 (-0.94, 0.4) 0.49
MSBB 219 -0.06 (-0.81, 0.74) 0.87
ROSMAP 822 -0.22 (-0.62, 0.19) 0.28
Meta-analysis Fixed Effects 1385 -0.2 (-0.51, 0.12) 0.22
Meta-analysis Random Effects 1385 -0.2 (-0.51, 0.12) 0.22
Heterogeneity I2 = 0.0%; Q-pval = 9.31e-01
Cohort N Beta P
Expanded Mayo Genotype 1977 0.93 (0.76, 1.1) 3.5e-26
Mayo 344 1.13 (0.71, 1.57) 2.4e-07
MSBB 219 0.47 (-0.02, 0.98) 0.06
ROSMAP 822 0.66 (0.38, 0.95) 5.2e-06
Meta-analysis Fixed Effects 3362 0.86 (0.72, 0.99) 6.5e-36
Meta-analysis Random Effects 3362 0.82 (0.6, 1.05) 1.3e-12
Heterogeneity I2 = 53.0%; Q-pval = 9.4e-02
Cohort N Beta P
Expanded Mayo Genotype 2001 1.45 (1.25, 1.66) 5.6e-44
Mayo 288 1.68 (1.15, 2.23) 9.1e-10
MSBB 135 1.58 (0.66, 2.7) 2.1e-03
ROSMAP 817 1.78 (1.34, 2.26) 1.8e-14
Meta-analysis Fixed Effects 3241 1.53 (1.35, 1.7) 1.4e-66
Meta-analysis Random Effects 3241 1.55 (1.34, 1.75) 1.2e-50
Heterogeneity I2 = 0.0%; Q-pval = 5.78e-01
Braak
Cohort N Beta P
Mayo 208 -0.39 (-1.06, 0.28) 0.25
MSBB 267 -0.06 (-0.58, 0.46) 0.83
ROSMAP 1091 -0.12 (-0.4, 0.16) 0.39
Meta-analysis Fixed Effects 1566 -0.14 (-0.37, 0.09) 0.23
Meta-analysis Random Effects 1566 -0.14 (-0.37, 0.09) 0.23
Heterogeneity I2 = 0.0%; Q-pval = 7.21e-01
Cohort N Beta P
Expanded Mayo Genotype 1922 0.75 (0.62, 0.88) 0.0e+00
Mayo 208 1.27 (0.82, 1.72) 4.3e-08
MSBB 267 0.3 (-0.05, 0.65) 0.09
ROSMAP 1091 0.56 (0.37, 0.75) 5.4e-09
Meta-analysis Fixed Effects 3488 0.69 (0.59, 0.79) 1.2e-42
Meta-analysis Random Effects 3488 0.7 (0.35, 1.04) 7.1e-05
Heterogeneity I2 = 77.8%; Q-pval = 3.59e-03
Cohort N Beta P
Expanded Mayo Genotype 1943 1 (0.87, 1.13) 0.0e+00
Mayo 208 1.6 (1.08, 2.13) 2.5e-09
MSBB 164 0.97 (0.39, 1.54) 1.0e-03
ROSMAP 1082 1.2 (0.95, 1.44) 0.0e+00
Meta-analysis Fixed Effects 3397 1.07 (0.96, 1.18) 1.8e-77
Meta-analysis Random Effects 3397 1.13 (0.92, 1.34) 4.6e-26
Heterogeneity I2 = 50.8%; Q-pval = 1.07e-01
Thal
Cohort N Beta P
Mayo 157 -0.08 (-0.8, 0.64) 0.83
Meta-analysis Fixed Effects 157 -0.08 (-0.8, 0.64) 0.83
Meta-analysis Random Effects 157 -0.08 (-0.8, 0.64) 0.83
Heterogeneity I2 = NA; Q-pval = NA
Cohort N Beta P
Expanded Mayo Genotype 1643 0.8 (0.64, 0.95) 0.0e+00
Mayo 157 1.64 (1.05, 2.23) 4.8e-08
Meta-analysis Fixed Effects 1800 0.85 (0.7, 1) 8.4e-29
Meta-analysis Random Effects 1800 1.17 (0.35, 1.99) 5.3e-03
Heterogeneity I2 = 86.4%; Q-pval = 6.58e-03
Cohort N Beta P
Expanded Mayo Genotype 1663 1.15 (0.98, 1.32) 0.0e+00
Mayo 157 2.3 (1.6, 3.01) 1.8e-10
Meta-analysis Fixed Effects 1820 1.22 (1.05, 1.38) 4.5e-47
Meta-analysis Random Effects 1820 1.68 (0.55, 2.8) 3.5e-03
Heterogeneity I2 = 89.6%; Q-pval = 1.93e-03
Age at Death
Cohort N Beta P
Mayo 344 0.72 (-1.57, 3.02) 0.54
MSBB 267 1.16 (-0.92, 3.24) 0.27
ROSMAP 1091 -0.17 (-0.84, 0.51) 0.63
Meta-analysis Fixed Effects 1702 0.02 (-0.6, 0.63) 0.96
Meta-analysis Random Effects 1702 0.13 (-0.7, 0.95) 0.76
Heterogeneity I2 = 0.0%; Q-pval = 4.01e-01
Cohort N Beta P
Expanded Mayo Genotype 1977 0.09 (-0.43, 0.61) 0.72
Mayo 344 -0.37 (-1.9, 1.17) 0.64
MSBB 267 -1.11 (-2.42, 0.2) 0.1
ROSMAP 1091 -0.53 (-0.97, -0.1) 0.02
Meta-analysis Fixed Effects 3679 -0.33 (-0.64, -9.8e-03) 0.04
Meta-analysis Random Effects 3679 -0.35 (-0.84, 0.14) 0.16
Heterogeneity I2 = 36.8%; Q-pval = 1.91e-01
Cohort N Beta P
Expanded Mayo Genotype 2001 0.17 (-0.36, 0.7) 0.53
Mayo 288 -0.9 (-2.79, 0.98) 0.35
MSBB 164 -0.92 (-2.88, 1.04) 0.36
ROSMAP 1082 -0.76 (-1.29, -0.23) 5.2e-03
Meta-analysis Fixed Effects 3535 -0.33 (-0.69, 0.03) 0.07
Meta-analysis Random Effects 3535 -0.42 (-1.09, 0.26) 0.22
Heterogeneity I2 = 55.2%; Q-pval = 8.27e-02
Beta

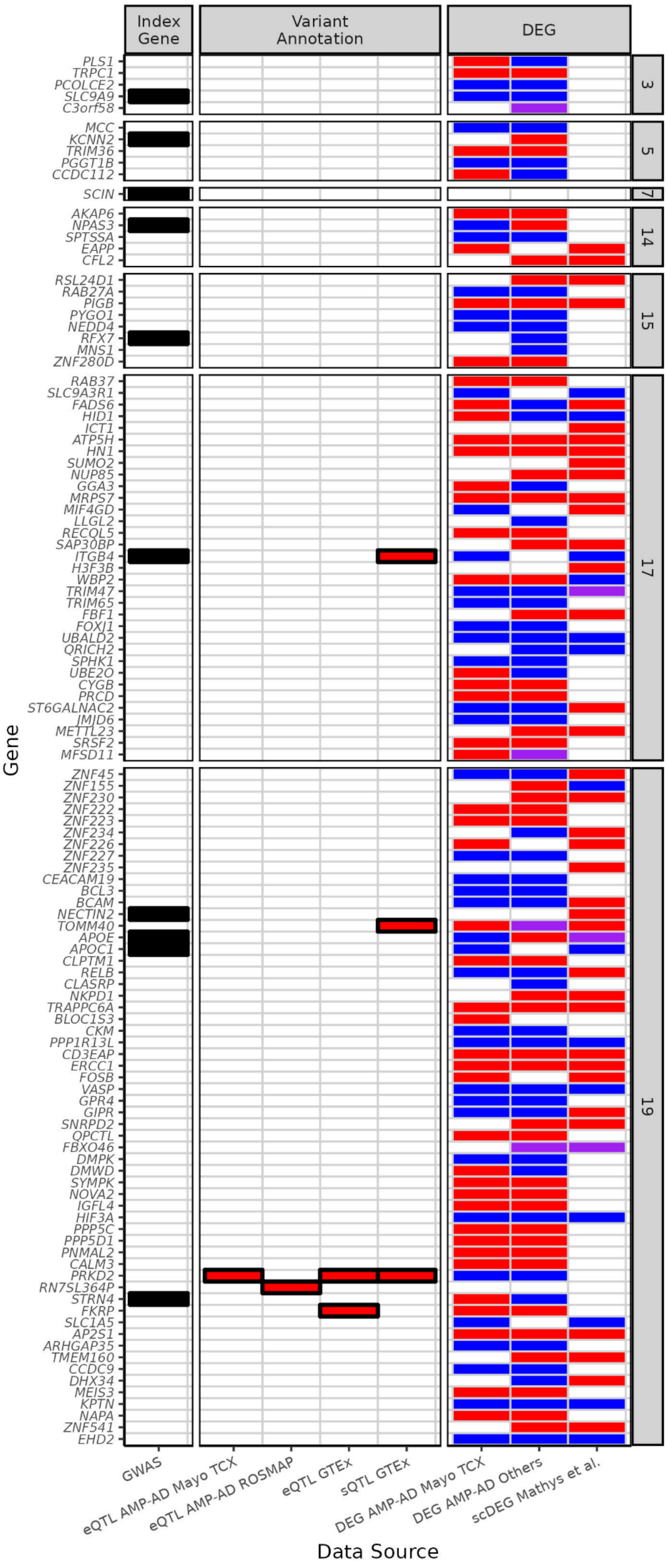

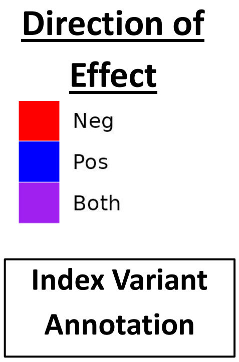

Figure S4: Summary of gene regulatory annotations for each GWS locus. Genes +/- 500Kb from each GWS variant as defined by Ensemble were investigated in multiple published datasets for dysregulation. Datasets include AMP-AD eQTL from the Mayo TCX and ROSMAP datasets, CNS related e/sQTL datasets from GTEx v8, and differential gene expression between ADs and non-ADs from the seven AMP-AD bulk RNAseq datasets (Mayo CER, four MSBB, and ROSMAP are combined into 'DEG AMP-AD Others') and Mathys et al 2019 single cell differential expression. Only genes with a study specific significant association are shown. Only associations significant in two or more AMP-AD datasets are shown unless there is additional evidence with that gene from another dataset. Association direction of effect is shown by fill color: red is positive, blue is negative, and purple means there were both positive and negative associations. Variant annotations to genes are outlined in black if the association involved the GWS index variant. As the *APOE, NECTIN2,* and *STRN4* loci overlap, these are shown together. Chromosome is on the right y-axis. DEG = Differentially Expressed Genes between ADs and nonADs; TCX = Temporal Cortex

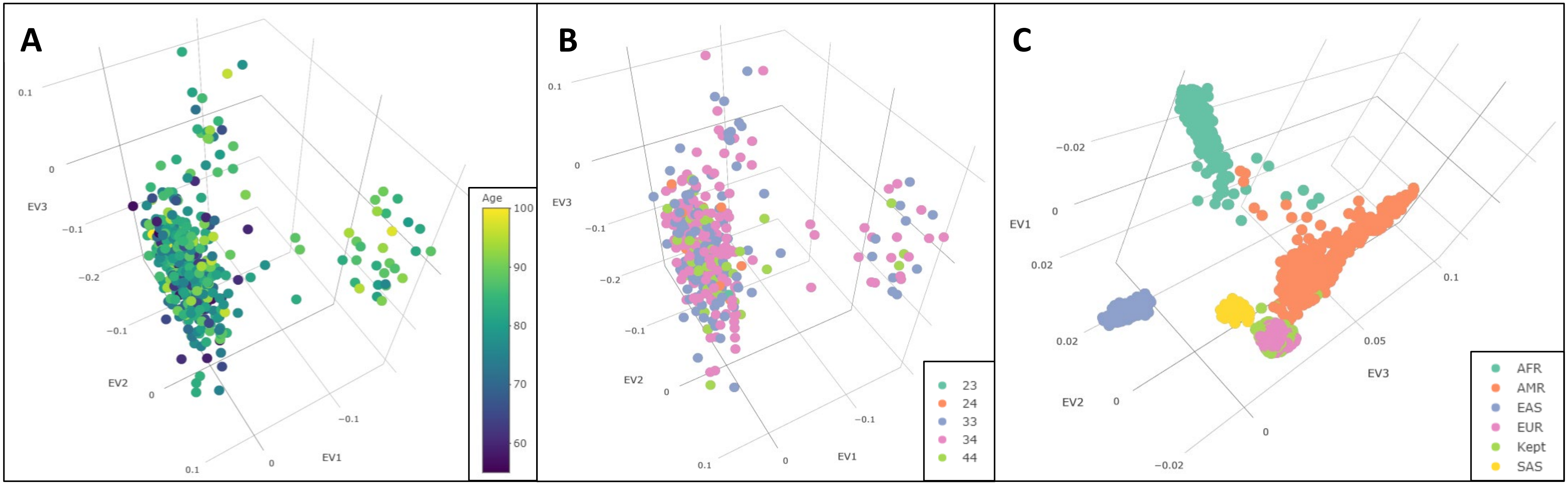

Figure S5: Population Substructure via Eigenstrat Analysis. Screenshots of the first three eigenvectors (EV1-3) plotted from the parent population (n=460) of our study cohort after analysis by Eigenstrat. **A:** Parent population colored by age at death. **B:** Parent population colored by *APOE* genotype. **C:** Superimposed view of study population (light green) with 1000 genomes populations.

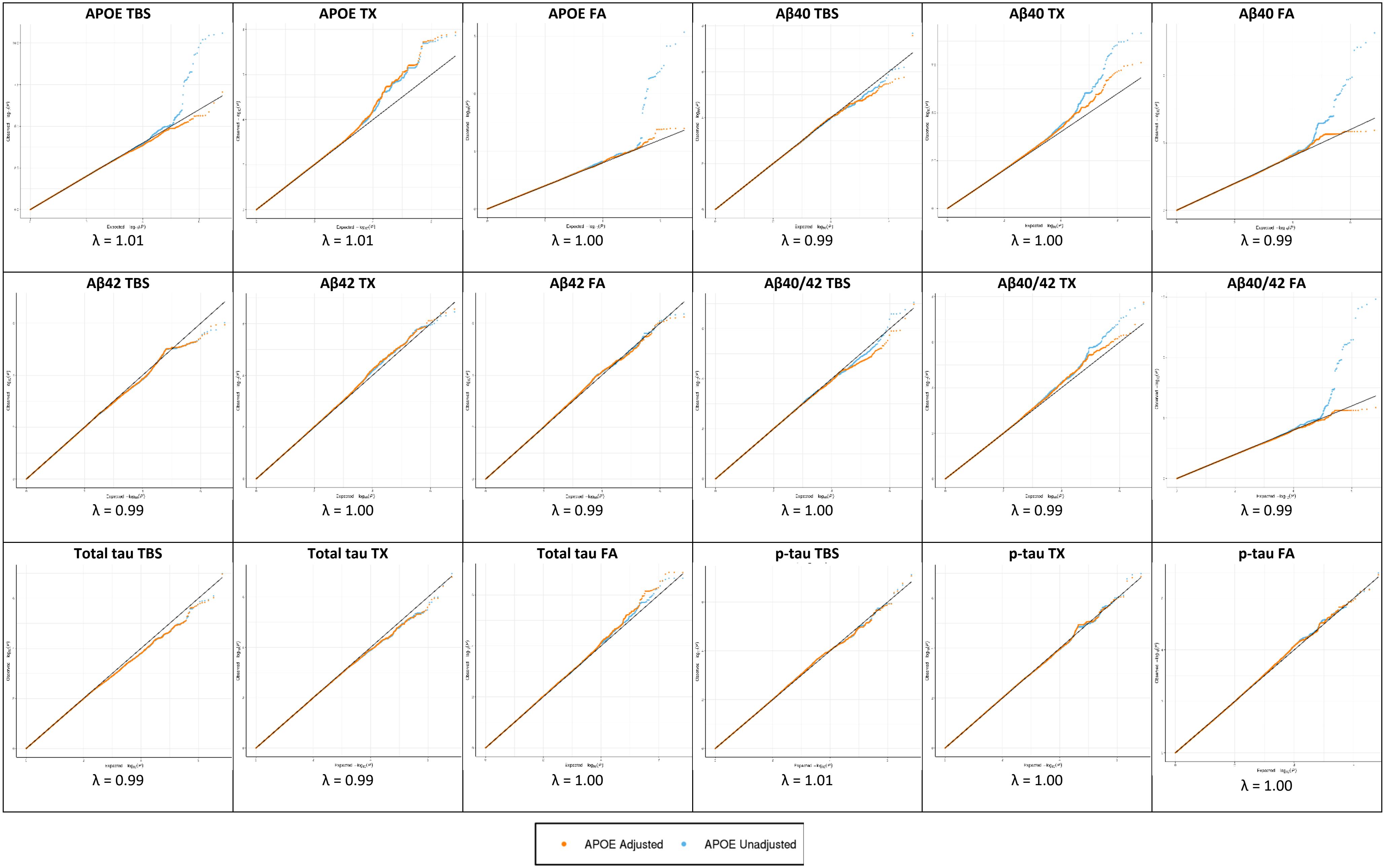

Figure S6: Quantile-Quantile (QQ) Plots of biochemical measure GWAS. QQ plots of each biochemical measure in *APOE-ε*2 and *APOE-ε*4 unadjusted (blue) and adjusted (orange) models. Genomic inflation values (λ) for *APOE* unadjusted models at the bottom of each plot. x-axis= expected –log10(p-value), y-axis = observed –log10(p-value).

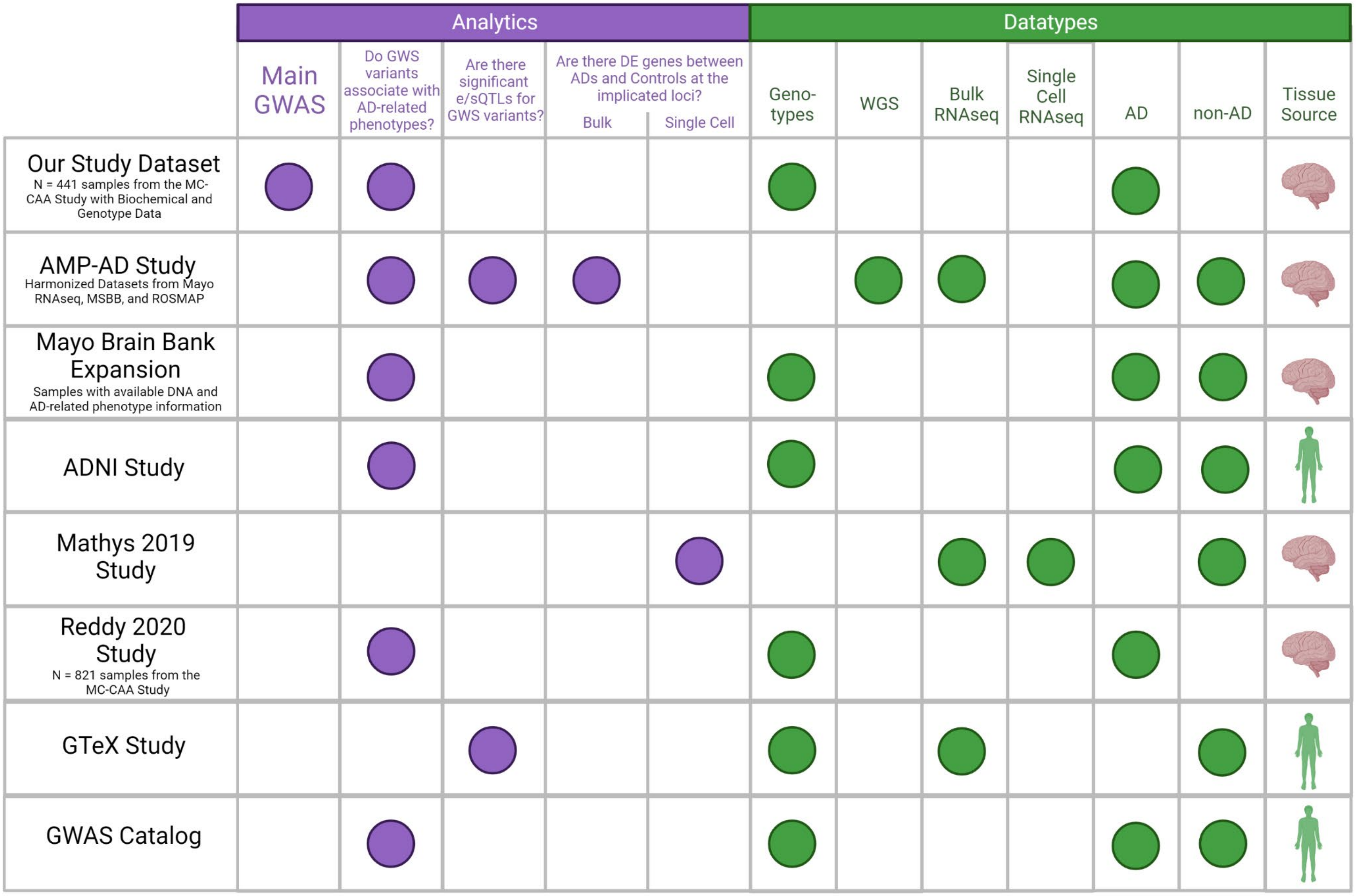

Figure S7: Datasets used for manuscript analytics. Description of the main datasets used for each analysis in this manuscript. Our study dataset is a subset of the larger MC-CAA study (also used in the Reddy et al 2020 study) as only N= 441 samples from this dataset had accompanying biochemical measures.
